# QTL pyramiding uncovers epistatic interactions that modify cuticle phenolics in tomato

**DOI:** 10.3389/fpls.2026.1863369

**Published:** 2026-08-03

**Authors:** Rida Barraj Barraj, María Urrutia, Juan Carlos Mateos del Amo, Ana González Moreno, Antonio Heredia, Rafael Fernández-Muñoz, Eva Domínguez

**Affiliations:** 1 Instituto de Hortofruticultura Subtropical y Mediterránea “La Mayora”, Universidad de Málaga-Consejo Superior de Investigaciones Científicas, Departamento de Mejora Genética y Biotecnología, Estación Experimental La Mayora, Málaga, Spain; 2 Instituto de Hortofruticultura Subtropical y Mediterránea “La Mayora”, Universidad de Málaga-Consejo Superior de Investigaciones Científicas, Departamento de Biología Molecular y Bioquímica, Universidad de Málaga, Málaga, Spain

**Keywords:** CHI, CHS, epistasis, phenolics, plant cuticle, Raman spectroscopy, tomato

## Abstract

The plant cuticle plays several roles with agronomic relevance such as controlling water loss or protecting against ultraviolet (UV) radiation and mechanical damage. In tomato fruit cuticle, phenolics are chiefly the flavonoid chalconaringenin and a small portion of phenolic acids. They confer the cuticle mechanical resistance as well as thermal properties and UV photoprotection. Nine QTLs related to the accumulation of cuticle phenolics in tomato fruit were previously detected in a *Solanum lycopersicum* × *S. pimpinellifolium* recombinant inbred population. Confocal Raman microscopy has been employed to separate the effect of these cuticle QTLs on the two phenolic fractions, showing that most QTL lines preferentially affected chalconaringenin accumulation, with only two QTL lines (*ph8.1* and *ph12.1*) having an equal effect on both chalconaringenin and phenolic acids. A QTL pyramiding strategy has been employed to study the combined effect of different *loci* and has revealed a complex array of interactions. Approximately 80% of the double and triple QTL lines displayed significant epistatic interaction. Notably, a QTL combination between chromosomes 5 and 12 that increases the percentage of cuticle phenolics beyond single QTL lines was identified. *CHALCONE SYNTHASE* (*CHS1* and *CHS2*) expression analyses have shown that *CHS1* seems to be the main enzyme responsible for the synthesis of chalconaringenin that is accumulated within the cuticle. Additionally, an *S. pimpinellifolium* genomic region in chromosome 5 that favors *CHALCONE ISOMERASE1* expression in the peel of ripening tomatoes without affecting chalconaringenin accumulation in the cuticle was identified, raising the possibility of an increase in fruit of downstream flavonols without compromising cuticle phenolics.

## 1 Introduction

At the plant surface, in contact with the environment, the cuticle plays numerous roles regarding plant protection against biotic and abiotic stresses. At the same time, it has an effect on fruit and vegetable quality affecting traits such as water loss and fungal growth during postharvest and, in tomato, fruit color and gloss (Adato et al., 2009; Parsons et al., 2012; Petit et al., 2013; Alkan and Fortes, 2015; Wu et al., 2023). The cuticle can be regarded as a modification of the cell wall, and it is chiefly composed of a polyester matrix named cutin, waxes, phenolics, and polysaccharides derived from the cell wall (Reynoud et al., 2022). Phenolics are mainly hydroxycinnamic acids, especially *p*-coumaric acid, which has been reported in all the cuticles analyzed so far and seems to be esterified to the cutin polyester (Rautengarten et al., 2012; Renault et al., 2017). Although the amount of cuticle phenolics tends to be low, in some species, they are present as a notable fraction (Kögel-Knabner et al., 1994; Reynoud et al., 2022; Kaplan et al., 2024; Manasherova et al., 2025). Additionally, in tomato fruit, the flavonoid chalconaringenin has also been identified within the phenolic fraction (Hunt and Baker, 1980). Tomato cuticle is characterized by the accumulation of phenolic acids (*p*-coumaric and *p*-hydroxybenzoic acids) throughout fruit development, while chalconaringenin only accumulates during ripening (España et al., 2014a, 2014b). Recently, confocal Raman microscopy (CRM) has been employed to identify spectral bands differentially assigned to chalconaringenin and phenolic acids and to study the topochemistry of phenolics within the tomato fruit cuticle (Reynoud et al., 2022; González Moreno et al., 2023). Thus, it was reported that during tomato fruit growth, phenolic acids are mainly located as a thin layer close to the outer side of the cuticle while, during ripening, phenolic acids and flavonoids are preferentially incorporated to the inner side of the cuticle (González Moreno et al., 2023).

The extensive work carried out in tomato cuticle has allowed several functions to be ascribed to the phenolic fraction. They have been shown to confer biomechanical resistance to the cuticle, especially during ripening when cell walls are being degraded (España et al., 2014a, 2014b). Additionally, incorporation of the yellow-orange colored flavonoid naringenin chalcone during ripening contributes to the characteristic red color of tomatoes (Adato et al., 2009; Ballester et al., 2010; España et al., 2014b). Cuticle enrichment in phenolics, rather than the amount of phenolics itself, has recently been shown to increase the glass transition temperature, modifying the thermal properties of the cuticle (Benítez et al., 2022, 2026). Finally, cuticle phenolics have been shown to provide photoprotection against ultraviolet-B (UV-B) in developing fruits and also UV-A in ripe fruits (Benítez et al., 2022; González Moreno et al., 2022). Hence, understanding the genetic basis behind natural variation for these compounds brings the possibility of improving several biophysical traits that have relevant agronomical implications including reduced cracking, improved photoprotection, or more biomechanically resistant cuticles under specific storage temperature conditions.

Numerous genes involved in the biosynthesis and regulatory network of cuticle deposition have been identified by means of reverse genetics (Yeats and Rose, 2013; Borisjuk et al., 2014), and more recently, the genetic basis behind the natural variability for some cuticle traits has been explored (Gosney et al., 2016; Ofner et al., 2016; Fernández-Moreno et al., 2017; Popovsky-Sarid et al., 2017; Rett-Cadman et al., 2019; Lin et al., 2020; Barraj Barraj et al., 2021; Yang et al., 2022). However, despite the relevance of phenolics in some cuticle roles of potential interest for plant breeding, only one work has addressed the analysis of the natural variability for this trait (Barraj Barraj et al., 2021). Additionally, phenolic compounds, especially flavonoids, are known for their health-promoting characteristics. Thus, in tomato, several studies have reported strategies to enhance the phenolic content of the fruit, thanks to the fact that the metabolic and regulatory pathways of phenolics are quite known (Muir et al., 2001; Davuluri et al., 2005; Butelli et al., 2008; Yang et al., 2022). However, although phenolic compounds are known to accumulate in the peel of tomato fruit, only in a few instances has the potential effect of these strategies at the cuticle level been studied. Thus, overexpression of the transcription factors *ROSEA* and *DELILA* in tomato fruit did not affect the phenolic content or the mechanical properties of the cuticle (España et al., 2014a). However, characterization of the mutation *colorless fruit epidermis* (*y*) responsible for pink tomatoes showed an almost absence of phenolics in the cuticle and a mechanically weaker cuticle (Adato et al., 2009). Similarly, silencing *CHALCONE SYNTHASE*, the enzyme responsible for the synthesis of chalconaringenin, or *ARLEQUIN/TOMATO AGAMOUS-LIKE1*, a transcription factor involved in fruit development, caused a significant reduction of cuticle phenolics and mechanical properties of the tomato fruit cuticle (España et al., 2014a; Giménez et al., 2015).

Quantitative traits are characterized by a complex genetic makeup involving several genes with small effects. Understanding how genes interact within a given genetic background and investigating how their accumulation affect the phenotype are crucial for plant breeding purposes. In a previous work, the combined analysis of recombinant inbred line (RIL) and introgression line (IL) populations between the domesticated tomato *Solanum lycopersicum* “MM” and a wild species, *Solanum pimpinellifolium*, accession “TO-937”, allowed the identification of nine additive QTLs and some epistatic effects related to the accumulation of phenolics in the tomato fruit cuticle, and a candidate gene analysis of some QTLs was carried out (Barraj Barraj et al., 2021). QTLs with a positive effect on cuticle phenolics were identified for both parents. The potential use of QTLs with desirable traits in a breeding program can strongly benefit from understanding the interactions between different QTLs in order to identify the most beneficial combinations. Therefore, in this work, epistatic interactions of phenolic-related loci were investigated by means of QTL pyramiding in double and triple QTL lines. This analysis will additionally improve our understanding of the pathways involved in phenolic accumulation within the tomato fruit cuticle.

## 2 Materials and methods

### 2.1 Plant material and QTL pyramiding

Eight lines harboring QTLs for the percentage of cuticle phenolics were selected from a 52 IL population derived from the recurrent backcross of *Solanum pimpinellifolium* L. “TO-937” (TO-937) into *Solanum lycopersicum* L. “Moneymaker” (MM) background (Barrantes et al., 2016). These lines had TO-937 introgressions for each of the QTLs (QTL lines), and the size of each introgression was reported in Barraj Barraj et al. (2021). Seven of these lines had a single cuticle phenolic QTL while the remaining one harbored two QTLs together (*ph7.1*+*ph7.2*). The eight lines were crossed among them to pyramid QTLs. Additionally, two subILs that separate *ph7.1* and *ph7.2* were also employed and crossed with the IL harboring the highest additive effect QTL, *ph1.1* (Barraj Barraj et al., 2021). Thus, a total of 30 F_1s_ were generated (**Supplementary Table 1**). A minimum of 20 F_1_ plants from each cross were grown in a polyethylene greenhouse and self-pollinated, and F_2_ seeds were collected. Between 100 and 200 plantlets from each F_2_ population were tested to identify plants combining TO-937 alleles for the different QTLs. DNAzol (Life Technologies, USA) was used to isolate DNA from these F_2_ populations. Flanking markers spanning each QTL, together with the peak marker, were reported in Barraj Barraj et al. (2021). Peak markers were used to design primers for plant selection (**Supplementary Table 2**). Single nucleotide polymorphisms (SNPs) were employed to select the desired combinations and resolved using high-resolution melting (HRM) analysis.

Forty plants of MM and TO-937 and 20 plants per QTL line (single, double, or triple) were grown in a polyethylene greenhouse at the Estación Experimental La Mayora, CSIC, Spain. Because of the high number of double and triple QTL lines, the study was divided and carried out in 3 years. Each year, MM, TO-937, and the single QTL lines were grown and studied together with a selected group of double and triple QTL lines. A random four-block design was employed to study the effect of cuticle phenolic QTL pyramiding. Flower trusses were vibrated twice a week to ensure that fruit set. Flowers were tagged at anthesis and fruits were harvested at three distinct physiological stages: immature green [15 days after anthesis (daa)], mature green (MG; 30–35 daa) and red ripe (RR; 55 daa). Sample size consisted of 100 fruits per genotype (200 for MM) at 15 daa and 40 fruits per genotype (80 in the case of MM) for the subsequent stages. Following harvest, all fruits were immediately processed for cuticle isolation. Additionally, a subset of fruits was collected at breaker stage (40–45 daa) and their epicarp tissue was collected and flash-frozen for gene expression analyses.

### 2.2 Cuticle isolation and characterization

Tomato fruits were halved and immersed in a 50 mM aqueous solution of sodium citrate (pH 3.7) with a mixture of 1% w/v cellulase and 1% w/v pectinase and 1 mM NaN_3_ to prevent microbial growth. Samples were incubated at 37°C with constant agitation. After 1 week, peels were detached from the rest of the fruit tissue and subsequently incubated in fresh solution to allow total enzymatic disruption of the remaining cellular tissues. After 7–10 days, the solution was refreshed and samples were incubated for at least another week. Isolated cuticles were air dried and stored under dry conditions. Cuticle extraction was carried out at three stages of development—immature green, MG, and RR—for MM, TO-937, and all the QTL lines (single, double, and triple).

The amount of cuticle was determined gravimetrically from flat pieces of cuticle of known surface area. Ten measurements per line, each corresponding to pieces of different RR fruits collected from different plants, were carried out. This analysis was carried out in MM, the single QTL lines that carried out overlapping QTLs for phenolics and amount of cuticle (*ph4.1*, *ph5.1*, *ph5.2*, and *ph12.1*) (Barraj Barraj et al., 2021), and the 22 double and triple QTL lines that contained them.

Cuticle color was analyzed with a Konica Minolta CR-400 colorimeter in the CIE 1976 L*a*b* and CIE L*C*h°color spaces using illuminant D65. Twenty-five pieces of cuticle were measured for TO-937 and single QTL lines, and 50 pieces of cuticle were measured for MM control. Each piece of cuticle corresponded to a different fruit collected from either 12 plants (TO-937 and single, double, and triple QTL lines) or 25 plants (MM). Additionally, CIE DE2000 color difference equation (Sharma et al., 2005) was employed to assess cuticle color differences of the double and triple QTL lines with the parental line MM.

Total cuticle phenolics were estimated at three stages of development (immature green, MG, and RR) using a UV–VIS spectrophotometer (Pharmacia Biotech, NJ, USA) after cutin depolymerization for 24 h in a 1% w/v of NaOH in methanol at 65°C with agitation (Domínguez et al., 2009). The fraction of phenolics present in the cuticle, expressed as percentage of cuticle phenolics, was estimated at each developmental stage from five biological samples per QTL line (single, double, and triple) and TO-937, whereas for MM control, 10 samples were studied. Each biological sample corresponded to pieces of cuticles isolated from at least five fruits collected from four different plants.

Raman spectroscopy analyses of isolated cuticles from MM, TO-937, and the individual QTL lines were carried out using an NRS 5100 (Jasco) Raman dispersive spectrometer coupled with a confocal microscope. The 785-nm laser was employed to minimize autofluorescence in the confocal Raman microscope. Samples were focused with a 100× objective [numerical aperture (NA) = 0.90]. A CCD detector cooled by the Peltier system was employed as detector. The laser power was set at 11.8 mW and the integration time was set to 30 s. Twenty scans were accumulated per sample. The internal side of isolated cuticles was analyzed since accumulation of flavonoids and phenolic acids during ripening has been shown to be differentially located across the tomato fruit cuticle (González Moreno et al., 2023). Band deconvolution and spectral analyses were carried out using the software OMNIC (Thermo Scientific, USA). Five to seven samples per line, each corresponding to a cuticle piece from a fruit collected from a different plant, were analyzed.

### 2.3 Expression analyses

RNA extraction from fruit epicarp at breaker stage was carried out with Trizol^®^ (Life Technologies, USA). Genomic DNA was removed by treating with RNase-free DNase, and RNA was cleaned with Nucleospin RNA clean-up (Macherey-Nagel, Germany). First-strand cDNA synthesis was carried out with the Super Script III First-Strand Synthesis Super Mix for qRT-PCR according to the manufacturer’s instructions (Invitrogen, USA). Three biological replicates corresponding to pools of fruit epicarp from five different plants were analyzed for TO-937 and the single QTL lines and six for MM. Relative transcript amount of the different genes was measured by RT-qPCR using SsoAdvanced™ SYBR^®^ Green Supermix (Bio-Rad, USA). The ΔΔCt method modified to account for primer efficiency and several endogenous genes (Vandesompele et al., 2002) was employed. The control genes employed were *CAC* (Solyc08g006960), *EXP* (Solyc07g025390), and *SAND* (Solyc03g115810) (Expósito-Rodríguez et al., 2008). For each biological replicate, three technical replicates were performed for MM, and TO-937 sequences of *CHS1* (Solyc09g091510), *CHS2* (Solyc05g053550), and *CHI1* (Solyc05g010320) were compared prior to primer design to avoid regions with mismatches. Primers employed and their efficiencies are presented in **Supplementary Table 3**.

### 2.4 Statistical analyses

IBM SPSS Statistics software was employed to carry out statistical analyses. *T*-tests were employed to compare the percentage of cuticle phenolics between the parental lines MM and TO-937. One-way analysis of variance (ANOVA) and Tukey-*b* tests were used to compare QTL lines and the parental line MM. Different letters indicate significant differences at *p* < 0.05. One-way ANOVA and Dunnett tests were employed to compare color and gene expression differences in TO-937 and single QTL lines against MM control. Two-way fixed-effects ANOVA with the two QTLs as main effects and MM and TO-937 homozygous alleles as the levels of those effects was employed to study the interaction between pairs of QTLs. For each combination, significance of the epistatic interaction was calculated as the *p*-value associated to the statistical double interaction between the two main effects. Also, two-way fixed-effects ANOVA was employed to study the effect of the interaction between genotype and season. Data are expressed as means ± standard error (SE).

## 3 Results

### 3.1 Effect of single QTL lines in the phenolic composition of the cuticle

**Figure 1:**
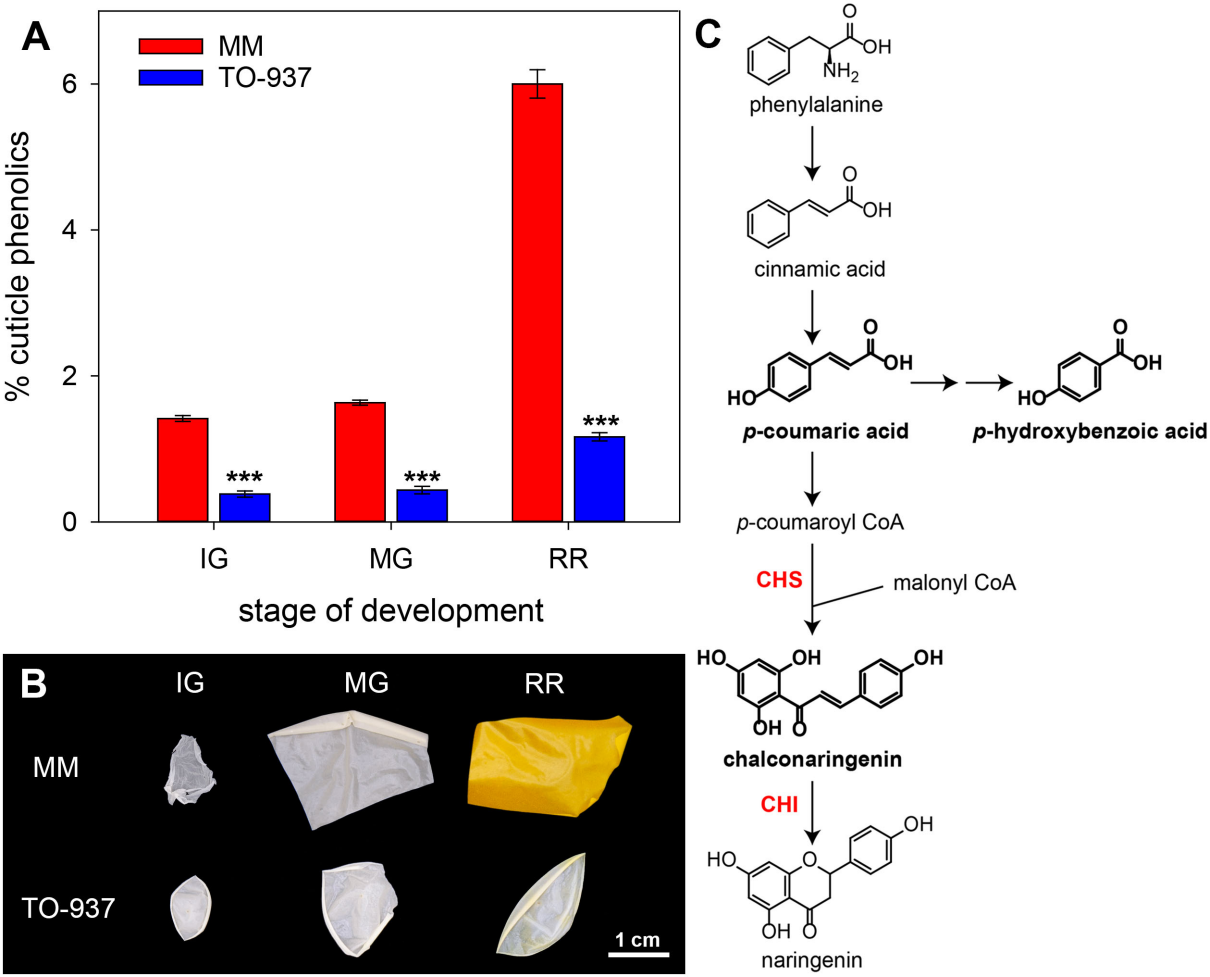
Cuticle phenolics in MM and TO-937. **(A)** Percentage of cuticle phenolics throughout development in both MM and TO-937. Asterisks indicate significant differences between lines (*p* < 0.001). Data presented as means ± SE. **(B)** Photograph of isolated fruit cuticles at different stages of development. **(C)** Biosynthetic pathway of phenolic compounds present in the cuticle. In bold, compounds identified in tomato fruit cuticle. In red, enzymes involved in flavonoid biosynthesis. CHS, CHALCONE SYNTHASE; CHI, CHALCONE ISOMERASE. IG, immature green; MG, mature green; RR, red ripe.

Comparison of parental lines showed that TO-937 exhibited a significantly lower percentage of cuticle phenolics throughout development (**Figure 1A**) (*p* = 4 × 10^−6^ immature green, *p* = 2 × 10^−5^ MG). These differences were exacerbated at RR (*p* = 5 × 10^−18^), when TO-937 cuticle remained almost colorless compared to the orange color observed in MM (**Figure 1B**). At early stages of development, the phenolic acids *p*-coumaric and *p*-hydroxybenzoic acids accumulate in the cuticle, while at RR, the flavonoid chalconaringenin is also incorporated, conferring the observed orange color to the cuticle of MM (**Figure 1C**; España et al., 2014a, 2014b). The colorless nature of the ripe cuticle of TO-937 (**Figure 1B**) can be considered an indication of little to no accumulation of chalconaringenin. In a previous work (Barraj Barraj et al., 2021), eight ILs harboring QTLs with a positive (*ph4.1*, *ph5.1*, *ph5.2*, and *ph12.1*) and a negative (*ph1.1*, *ph7.1 + 7.2*, *ph8.1*, and *ph12.2*) effect on percentage of cuticle phenolics were identified. However, cuticle phenolics include phenolic acids and chalconaringenin, which can only be fully extracted from the cuticle after cutin depolymerization. The harsh chemical conditions needed to depolymerize the cutin matrix causes chalconaringenin degradation, preventing its individual quantitation (España et al., 2014b). Since there is also an increase in phenolic acids during ripening, the difference in phenolic compounds between MG and RR cannot be considered a proxy for the chalconaringenin fraction. Thus, QTLs identified at RR could be associated with changes in either phenolic acids or chalconaringenin, or both. To overcome this limitation, CRM was employed to study the cuticles of the parental and single QTL lines.

**Figure 2:**
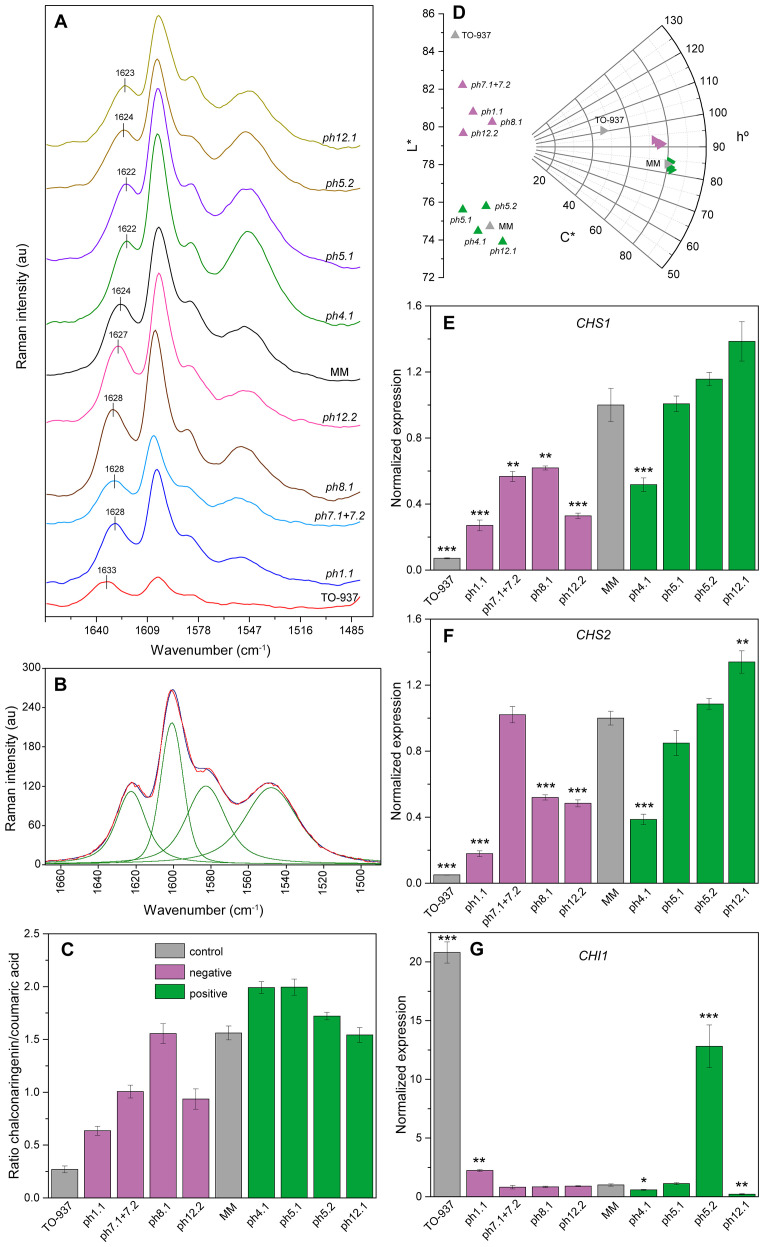
Characterization of the phenolic fraction of the parental lines and the single QTL-lines. **(A)** Raman spectra, within the 1670-1480 cm^-1^ region, of isolated cuticles at red ripe. **(B)** Deconvolution of this spectral region displaying the contribution of four bands. **(C)** Ratio of the band area assigned to chalconaringenin (~1550 cm^-1^) and phenolic acids (~1620 cm^-1^). **(D)** Color parameters, L* (luminosity), C* (chroma) and hº (hue), of isolated cuticles at red ripe. **(E–G)** Expression levels, normalized to MM, of genes involved in the synthesis of flavonoids in the epicarp of tomatoes at breaker. Asterisks (* *p* < 0.05; ** *p* < 0.01; *p* < 0.001) indicate significant differences with MM. All QTL-lines were statistically different from TO-937. In grey parental lines MM and TO-937, in purple/green lines harboring QTLs with negative/positive effect on cuticle phenolics. Data are expressed as means ± SE. *CHS, CHALCONE SYNTHASE; CHI, CHALCONE ISOMERASE*.

**Figure 2A** shows Raman spectra within the 1,680–1,480 cm^−1^ region of RR isolated cuticles of parental lines together with the lines harboring QTLs for the percentage of cuticle phenolics. Deconvolution of this spectral region identified the contribution of four bands (**Figure 2B**), and their assignations according to the literature are shown in **Supplementary Table 4**. The band around 1,620 cm^−1^ was assigned to a phenolic fraction enriched in phenolic acids, mainly *p*-coumaric acid, while the 1,550 cm^−1^ band was associated to chalconaringenin (González Moreno et al., 2023). The other two bands, around 1,585 and 1,602 cm^−1^, could be assigned to the interaction between phenolic compounds, either phenolic acids or chalconaringenin. A spectral shift in the band assigned to phenolic acids was observed between parental lines, while in MM, this band was present at 1,624 cm^−1^, and in TO-937, it was located at 1,633 cm^−1^. Interestingly, in the lines with QTLs for reduced percentage of cuticle phenolics (*ph1.1*, *ph7.1 + 7.2*, *ph8.1*, and *ph12.2*), located in the graph between TO-937 and MM, this band displayed an intermediate position between the parental lines, whereas in the lines with QTLs with a positive effect (*ph4.1*, *ph5.1*, *ph5.2*, and *ph12.1*, located above MM in the graph), the band showed the same position as MM (**Figure 2A**). This band shift is associated with changes in the chemical environment. **Figure 2C** shows the ratio between the areas of the 1,550 and 1,620 cm^−1^ bands (*A*_1550_/*A*_1620_), which can be considered an indication of the chalconaringenin/phenolic acids ratio. TO-937 exhibited an outstanding sixfold reduction of this ratio compared to MM. This low ratio, combined with the reduced percentage of total phenolics (**Figure 1**), indicates a preferential decrease in chalconaringenin, with phenolic acids being the main contributors to the phenolic domain present in the cuticle of TO-937. In a similar fashion to TO-937, QTL lines with a negative effect on cuticle phenolics, *ph1.1*, *ph7.1 + 7.2*, and *ph12.2*, showed a lower *A*_1550_/*A*_1620_ ratio compared to MM. On the other hand, QTL lines with a positive effect on cuticle phenolics, *ph4.1*, *ph5.1*, *and ph5.2*, displayed a higher *A*_1550_/*A*_1620_ ratio compared to MM, which is indicative of a preferential accumulation of chalconaringenin over phenolic acids. Interestingly, QTL lines *ph8.1* and *ph12.1*, which harbor, respectively, QTLs with a negative and a positive effect on the percentage of total phenolics, showed *A*_1550_/*A*_1620_ ratios similar to MM. This indicates a proportional reduction (*ph8.1*) and increase (*ph12.1*) of phenolic acids and chalconaringenin.

Chalconaringenin confers orange color to the tomato fruit cuticle. Cuticle color analyses of the parental and single QTL lines showed remarkable differences in all color parameters: lightness (L*), saturation (chroma, C*), and tone (hue, h°) (**Figure 2D**, **Supplementary Table 5**). TO-937 cuticle color exhibited increased L* and h° and reduced C* compared to MM. The differences in color saturation and tone could be attributed to the differential accumulation of chalconaringenin, while the lightness increase could be partially a consequence of the lower amount of cuticle (Barraj Barraj et al., 2021). Similarly, all single QTL lines with reduced percentage of total phenolics showed a significant decrease (*p* < 0.001) in color saturation (C*) and a yellower tone (higher h° values). However, of the lines with QTL for enhanced cuticle phenolics, only *ph4.1* displayed a significant effect on cuticle color, with increased saturation (*p* = 0.001) and lower hue (more orange) (*p* = 0.0004) than MM.

Chalconaringenin accumulation in ripe tomatoes is due to the combination of enhanced *CHALCONE SYNTHASE* (*CHS*) expression during ripening together with *CHALCONE ISOMERASE* (*CHI*) expression below the limit of detection, thus limiting the accumulation of downstream flavonoids (Muir et al., 2001). Indeed, two *CHS* genes (*CHS1* and *CHS2*) have been reported to be expressed in tomato epicarp and their expressions related to chalconaringenin incorporation to the cuticle of ripe tomatoes (España et al., 2014a). Although none of these genes locate within any of the QTL regions, the reported changes in total phenolics indicate that their expressions may be altered in some of the QTL lines. Thus, *CHS* and *CHI* expression levels were analyzed in the epicarp of fruits at breaker stage (**Figures 2E–G**). Comparison of parental lines showed a significant reduction of *CHS1* (*p* = 9 × 10^−17^) and *CHS2* (*p* = 2 × 10^−18^) expression in TO-937 compared to MM (**Figures 2E, F**; **Supplementary Figure 1**). Out of the four QTL lines with a positive effect on total phenolics, only *ph12.1* showed expression level of both *CHS* genes higher than MM, in agreement with the increased percentage of total phenolics reported for this line (Barraj Barraj et al., 2021). Despite the fact that *ph4.1* also increased total cuticle phenolics, expression levels of *CHS1* (*p* = 3 × 10^−4^) and *CHS2* (*p* = 9 × 10^−8^) were significantly lower than MM. The remaining positive-effect QTLs, *ph5.1* and *ph5.2*, showed values similar to MM. On the other hand, QTL lines with a negative effect on total phenolics displayed *CHS* expression lower than MM. The lowest values were detected in *ph1.1*, the QTL with the highest additive effect, and the line that displayed the highest reduction of chalconaringenin/phenolic acid ratio (**Figure 2D**). Although slight changes between *CHS1* and *CHS2* expression were observed in several QTL lines, in general, both *CHS* genes exhibited a similar behavior, with the exception of *ph7.1 + 7.2*, where *CHS1* expression was significantly reduced (*p* = 0.002) while *CHS2* expression remained similar to MM.

Regarding *CHI1*, its expression during ripening was extremely low in MM, especially in comparison to *CHS1* and *CHS2* (**Supplementary Figure 1**), as expected from the literature (Muir et al., 2001), but a surprising 22-fold increase was observed in TO-937 (**Figure 2G**). *CHI1* expression in the QTL lines showed values similar to MM or lower. However, a remarkable 13-fold increase was detected in *ph5.2*, similar to the high *CHI1* expression detected in TO-937 (**Figure 2G**).

### 3.2 Interaction between QTLs for percentage of cuticle phenolics

**Figure 3:**
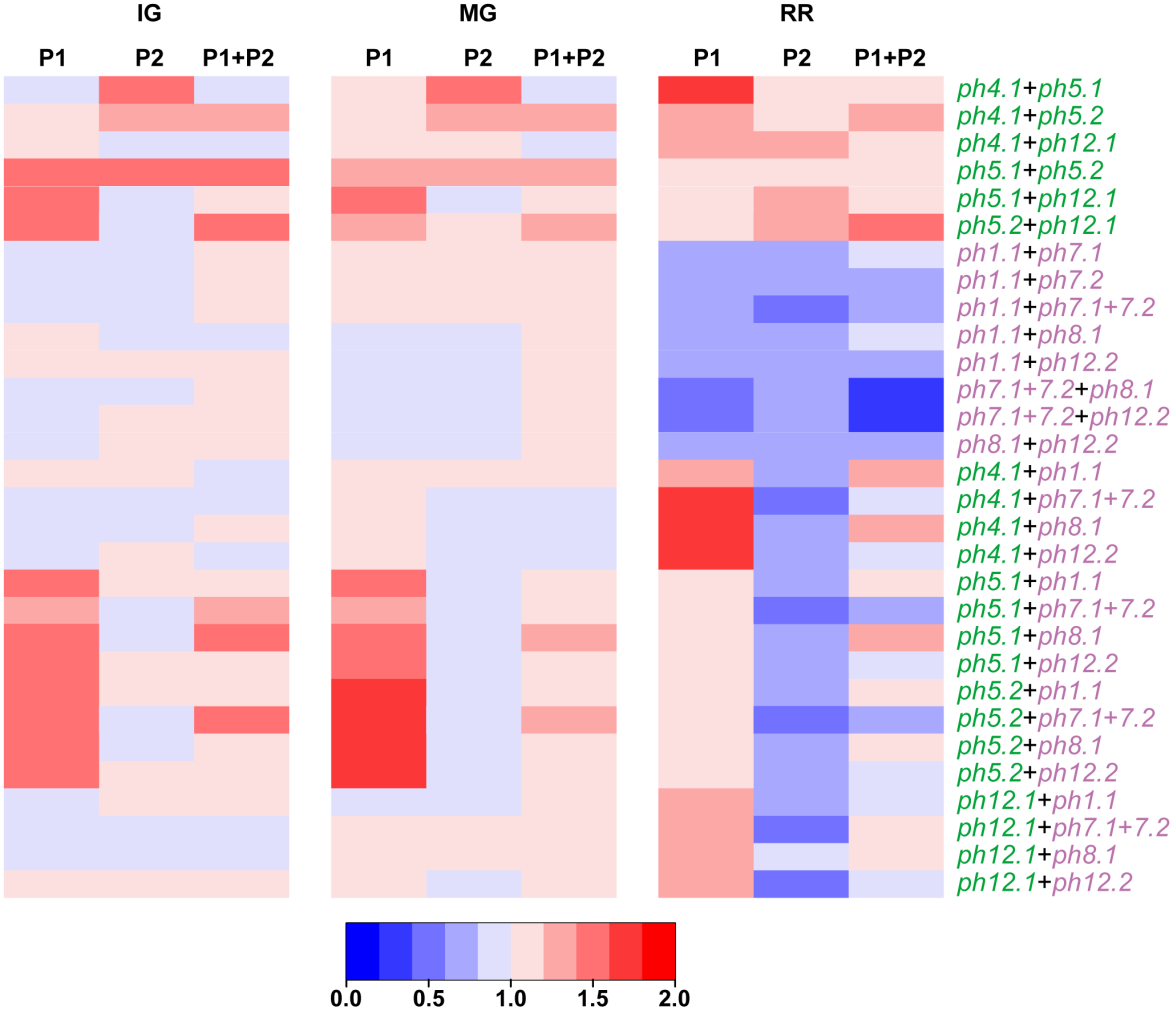
Heat map of the values of percentage of cuticle phenolics for the single, double, and triple QTL lines. Data are shown as fold change relative to MM control. IG, immature green; MG, mature green; RR, red ripe; P1 and P2, single or double (case of *ph7.1 + 7.2*) QTL lines; P1+P2, double or triple (case of *ph7.1 + 7.2*) QTL lines. In purple/green, QTLs with a negative/positive effect on phenolic accumulation.

Thirty double and triple QTL lines were obtained through crossing single QTL lines followed by marker-assisted selection (**Supplementary Table 1**). Six and eight of them corresponded to combinations of QTLs with a positive and a negative effect, respectively, while 16 lines accumulated QTLs with an opposite effect (positive and negative) on cuticle phenolics. **Figure 3** shows the percentage of cuticle phenolics at three stages of development for all the double and triple QTL lines, and their corresponding single QTL lines, expressed as fold changes relative to MM control. At RR, only one of the six lines pyramiding QTLs with a positive effect, *ph5.2 + 12.1*, showed a notable increase in cuticle phenolics compared to MM and the respective parental QTL lines. Of the eight lines pyramiding QTLs with a negative effect on cuticle phenolics, the lowest percentage was obtained with the triple QTL lines *ph7.1 + 7.2 + 8.1* and *ph7.1 + 7.2 + 12.2*. It is surprising that *ph1.1*, despite having the highest additive effect in the RIL population, not only showed a small effect in the IL population (Barraj Barraj et al., 2021), but also had little contribution to cuticle phenolics in the double and triple lines pyramiding negative effect QTLs. The negative effect of *ph1.1* was only observed in the combination *ph1.1 + 12.1*, where *ph1.1* reduced the positive effect of *ph12.1*.

**Figure 4:**
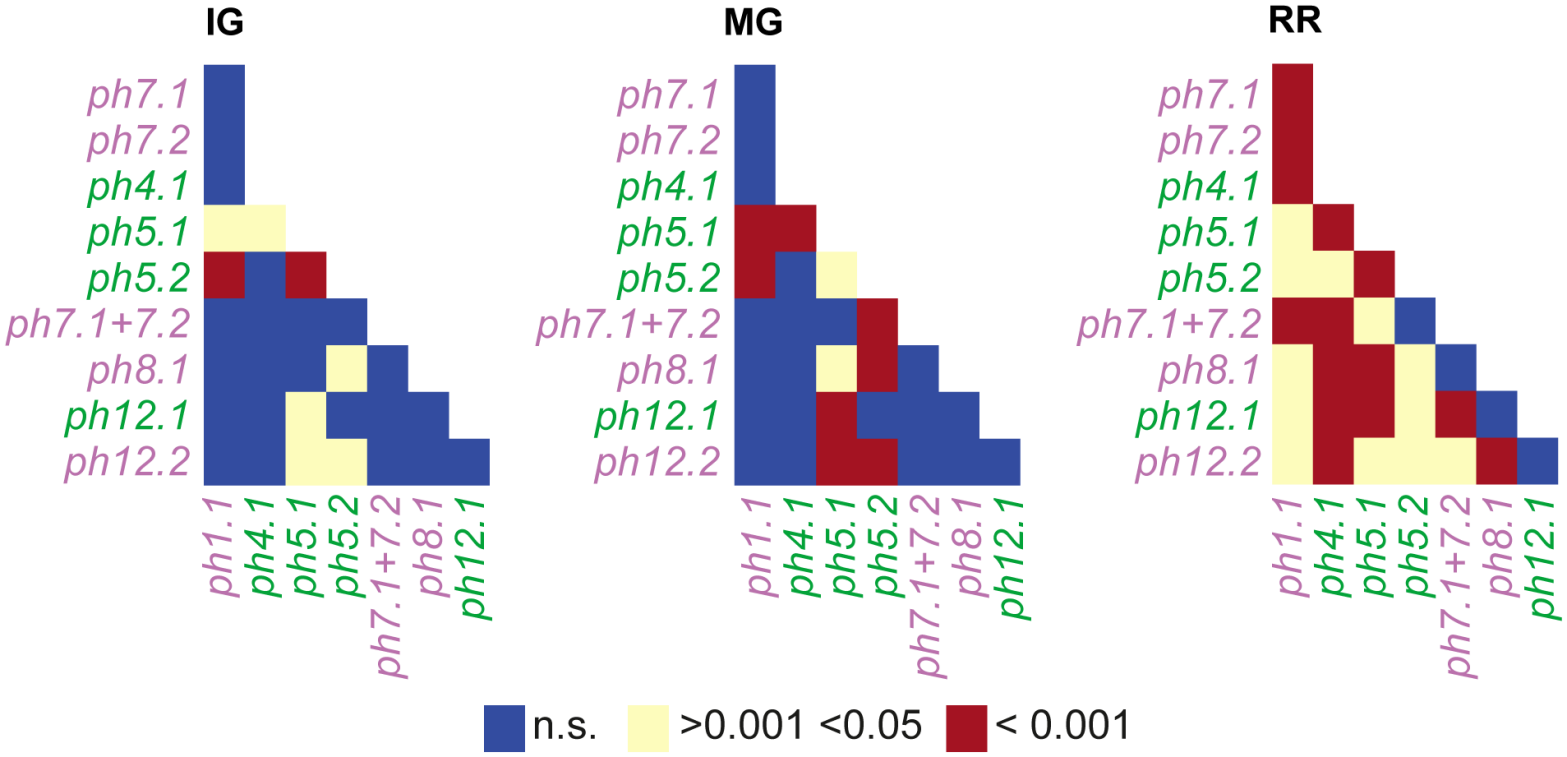
Heat map of *p*-values associated with epistatic interaction. *p*-values (calculated by two-way ANOVA) are presented for percentage of cuticle phenolics at three stages of tomato fruit development in all the double and triple QTL lines. IG, immature green; MG, mature green; RR, red ripe. In purple/green, QTLs with a negative/positive effect on phenolic accumulation.

The above-mentioned results point to little additive effect and the presence of epistatic interactions between these QTLs. **Figure 4** shows a heat map of the probability values of epistatic interactions for each of the double and triple QTL lines. As it can be clearly observed, at stages prior to ripening, most of the crosses involving *ph5.1* and/or *ph5.2* showed significant epistatic interactions, whereas the other QTL lines showed no epistatic interaction. At RR, however, over 86% of the double and triple QTL lines studied displayed a significant epistatic interaction. These results show that variation in cuticle phenolics is governed not only by additive QTL effects but also by strong non-additive interactions.

**Figure 5:**
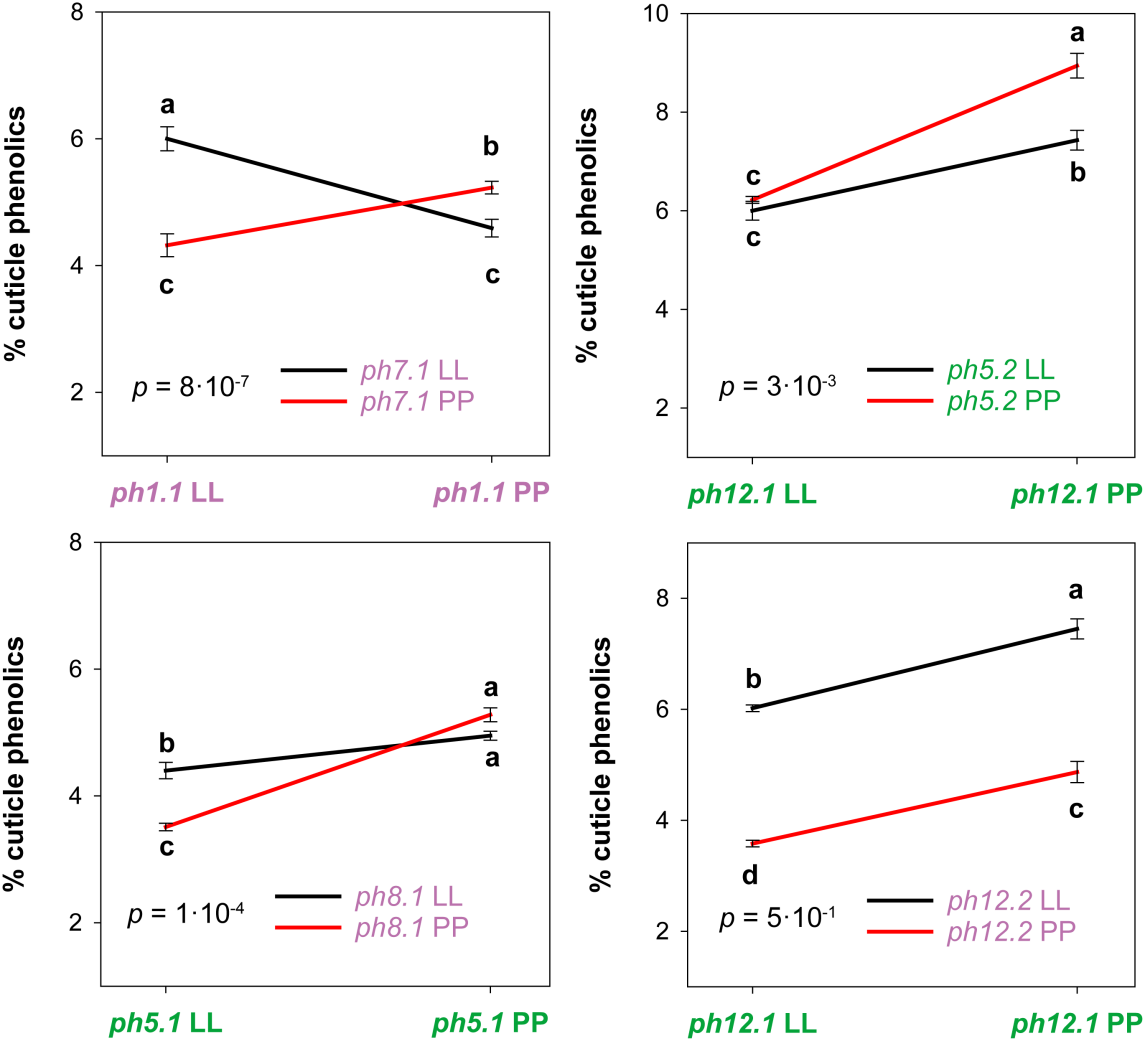
Examples of epistatic interactions. Interactions between QTLs with negative, positive, and positive × negative effects on cuticle phenolics at red ripe are shown. Data presented as means ± SE. Letters indicate significant differences among lines harboring different QTLs (*p* < 0.05). Significance of epistatic interaction is also given as *p*-values calculated by two-way ANOVA. In purple/green, QTLs with negative/positive effect on phenolic accumulation.

**Figure 5** shows examples of the different interactions detected in the double and triple QTL lines. With the exception of *ph7.1 + 7.2*+*ph8.1*, with only additive effect, all the lines pyramiding QTLs for reduced cuticle phenolics showed epistatic interactions (**Supplementary Figure 2**). The triple QTL line *ph7.1 + 7.2*+*ph12.2* displayed a combination of epistatic and additive effects, and the reduction in phenolics caused by *ph12.2* was more evident when *ph7.1 + 7.2* were present as MM alleles, as it can be seen by the different slopes. Epistatic interactions were observed between *ph12.2* and *ph1.1* or *ph8.1*, with the double QTL line showing similar values to *ph12.2* in both cases. Accumulation of *ph1.1* with *ph8.1* or *ph7.2* did not have an effect on cuticle phenolics, since the percentage of cuticle phenolics was similar to both parental lines. However, interaction of *ph7.1* with *ph1.1* showed that the double QTL line increased its percentage in phenolics compared to the single QTL lines, but without reaching the values of MM control. A similar effect was observed in the triple QTL line *ph1.1*+*ph7.1 + 7.2*, but in this case, percentage of cuticle phenolics was intermediate between parental QTL lines.

Regarding lines pyramiding QTLs with a positive effect on cuticle phenolics, only *ph5.1*+*ph5.2* showed no effect. This was expected, since these single QTL lines only showed effect at stages prior to RR (**Supplementary Figure 3**). However, they did show epistatic interaction with *ph12.1* and *ph4.1* modifying their effects. Thus, *ph5.1* interacted with *ph4.1* and *ph12.1* cancelling their effects. A similar behavior was observed in the interaction between *ph4.1* and *ph12.1*, with the double QTL line displaying a lower percentage of cuticle phenolics than *ph4.1.* Conversely, *ph5.2* did not modify the effect of *ph4.1* and had a positive effect increasing the percentage of cuticle phenolics in combination with *ph12.1*.

Out of the 16 lines pyramiding QTLs with positive and negative effects on cuticle phenolics, 13 showed epistatic interactions (**Supplementary Figures 4**, **5**). Double QTL lines *ph12.1*+*ph8.1* and *ph12.1*+*ph12.2* only displayed additive effects, with the former cancelling their opposite effects and resulting in values similar to MM and the latter displaying values intermediate between the single QTL lines, but lower than MM control. Similarly, a percentage of cuticle phenolics intermediate between parental QTL lines was observed in six other combinations (*ph4.1+ph7.1 + 7.2*, *ph5.1+ph7.1 + 7.2*, *ph12.1+ph7.1 + 7.2*, *ph4.1+ph8.1*, *ph4.1+ph12.2*, and *ph5.2+ph12.2*) with the double/triple QTL lines displaying values similar to or above MM control. The triple QTL line *ph5.2*+*ph7.1 + 7.2* showed values similar to *ph7.1 + 7.2*, indicating that *ph5.2* did not modify the effect of the negative QTL. An opposite behavior was observed in the double QTL lines pyramiding negative effect QTLs *ph1.1* and *ph8.1* with positive effect QTLs *ph5.1* and *ph5.2*, as well as in *ph12.2*+*ph5.1* and *ph1.1*+*ph4.1*. In all of these double lines, the negative effects of the single QTL were canceled. The percentage of cuticle phenolics was similar to the parental QTL line with positive effects.

Analysis of epistatic interactions for cuticle phenolics at IG and MG in the double and triple QTL lines involving *ph5.1* and *ph5.2* are presented in **Supplementary Figures 6**-**9**. With a few exceptions, the positive increase in phenolics caused by *ph5.1* or *ph5.2* was cancelled at both immature green and MG by the other QTLs.

The four QTLs that increase the percentage of cuticle phenolics overlapped to some extent with QTLs related to the amount of cuticle (Barraj Barraj et al., 2021). In three cases (*ph4.1*, *ph5.1*, and *ph12.1*), the associated QTL for the amount of cuticle (*cm*) showed an opposite effect to that observed for percentage of cuticle phenolics (**Supplementary Figure 10**). Therefore, it was possible that the increase in percentage of cuticle phenolics in these single QTL lines was caused by a reduction in the amount of cuticle. However, determination of the amount of cuticle in the double and triple QTL lines involving these four QTLs showed no clear relationship between the amount of cuticle and percentage of cuticle phenolics (**Supplementary Figure 11**).

As was mentioned previously, chalconaringenin is responsible for the orange color of the ripe tomato cuticle. Cuticle color was measured in MM and the double and triple QTL lines in order to determine color changes with respect to MM control using the CIE DE2000 (ΔE) equation. A clear relationship was observed between ΔE and fold change (relative to MM) in the percentage or amount of cuticle phenolics (**Supplementary Figure 12**). In both cases, but especially for the amount of phenolics, data were grouped following two different lines. As the relative amount or percentage of cuticle phenolics increased approaching 1 (values similar to MM), the differences in color decreased. The second group of data showed that as cuticle phenolics continued to increase (values higher than MM), color differences increased again. This is a clear indication that phenolics, especially their amount, influence the color of tomato fruit cuticle. The highest color differences of the double/triple QTL lines corresponded to negative × negative interactions, specifically those that caused the highest reduction in phenolics. Positive × positive interactions also caused color change, but it was not as manifest as the negative effects.

## 4 Discussion

### 4.1 Deciphering the complexity behind the QTLs for cuticle phenolics

Numerous strategies are currently available for dissecting the genetic basis behind phenotypic variation within natural populations. However, phenotypic variability is often quantitative, with several genes with variable effects involved, influenced by the environment and genetic factors. In this sense, cuticle biosynthesis has been reported to be modified by environmental conditions, as well as phenolic accumulation in plants (Hull et al., 1975; Cohen and Kennedy, 2010). Although genotype × environment interactions can influence phenotype (Cooper et al., 2009), results of the parental and single QTL lines showed similar effects within seasons (**Supplementary Table 6**) and were consistent with those previously reported (Barraj Barraj et al., 2021). A genotype × season interaction analysis showed a significant effect of the environment on the percentage of cuticle phenolics between winter and summer (*p* = 10^−6^), while no differences were detected between both summer seasons. However, this study across seasons was not carried out with the double and triple QTL lines. Thus, the epistatic effects reported here should be validated on different seasons prior to its use in breeding programs. It is worth mentioning that the percentage of cuticle phenolics did not change between seasons in TO-937 (**Supplementary Table 1**). This could be indicative of a low, constitutive level of cuticle phenolics, or that the effect of environmental conditions can vary among cultivars and/or in red-fruited wild species. Given the impact of climate change on crop growth and yield, this is a topic that deserves further study.

Many analyses of natural variability focus on major effects, yet, uncovering epistatic (non-additive) interactions is important for plant breeding since it allows the identification of the most beneficial interactions that produce a desirable phenotype within a given genetic background (Dwivedi et al., 2024). In the last years, numerous works have highlighted the importance of epistasis in traits such as inflorescence branching, plant height, flower sex determination, or metabolite accumulation (Shen et al., 2014; Soyk et al., 2017; Zebell et al., 2025; Zunjare et al., 2025). In this work, of the 30 double/triple QTL lines studied, only 7 (3 positive × positive and 4 negative × negative combinations) did not show an effect on the percentage of cuticle phenolics due to QTL accumulation, displaying values similar to the single QTL lines. Regarding epistasis, 80% of the studied crosses exhibited some degree of epistatic interaction, which is well above the only one previously identified using specialized software (Barraj Barraj et al., 2021). Moreover, the two QTLs with the highest percentage of variance within the RIL population, *ph1.1* and *ph8.1*, only displayed a small reduction in cuticle phenolics in the IL population (Barraj Barraj et al., 2021), revealing epistatic interactions within the genetic background. These results underscore the practical implications of these analyses for crop engineering through the identification of advantageous genetic combinations and deepen our understanding on genetic interactions yielding similar phenotypic effects.

Cryptic genetic variation can have considerable effect on a phenotype under specific environmental conditions or different genetic backgrounds (Gibson and Dworkin, 2004). It is interesting to note that, although TO-937 displayed a very low percentage of phenolics within the cuticle, four QTLs with a positive effect of the TO-937 allele were identified (Barraj Barraj et al., 2021). However, two of these QTLs, *ph5.1* and *ph5.2*, when placed in the MM background did not show any effect on cuticle phenolics at the RR stage, only at previous stages of development, leading to the idea that their effect might solely be attributed to minor changes in cuticle phenolics during growth, without a significant effect during ripening (Barraj Barraj et al., 2021). However, Raman spectroscopy analysis of *ph5.1* and *ph5.2* cuticles at RR showed an altered ratio of chalconaringenin/phenolic acid band ratio, which, together with the increased *CHI1* expression level at breaker in *ph5.2*, is indicative of an effect on cuticle phenolics despite not being identifiable as percentage of cuticle phenolics at RR. Moreover, the combination of *ph5.1* and *ph5.2* with other QTLs allowed their effects on cuticle phenolics to be manifested, but only in specific genetic combinations. This points out to a negative interaction between MM background and *ph5.1* and *ph5.2* TO-937 alleles that buffered their effects.

**Figure 6:**
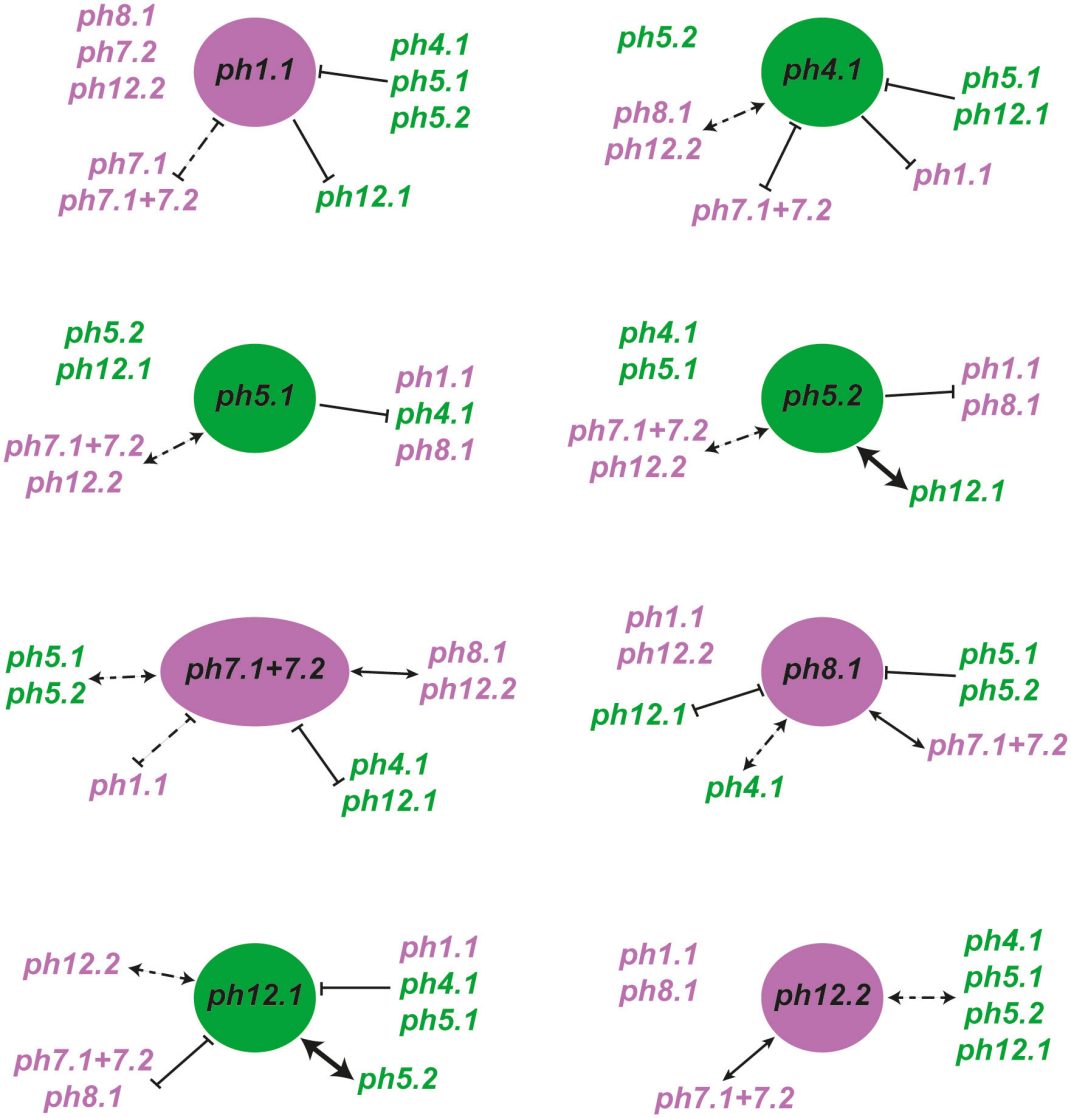
Summary of the interactions identified between TO-937 alleles of the QTLs for cuticle phenolics. In purple/green, QTLs with a negative/positive effect on phenolic accumulation. Dashed lines indicate an intermediate effect in the combined lines; sharp arrow lines represent an interaction that causes a decrease (regular line) or increase (bold line) in the double homozygous compared to the parental lines; blunt arrow lines indicate an inhibitory effect of one QTL toward the other; double blunt lines indicate that QTLs cancelled each other; dashed double blunt lines indicate a partial inhibition of both QTLs, with values higher than the individual lines, but without reaching those of MM control; no lines connecting QTLs indicate no interaction, with the double homozygous displaying values similar to the parental line with the lowest (in the case of a negative × negative interaction) or highest (in the case of a positive × positive interaction) percentage of cuticle phenolics.

**Figure 6** summarizes the interactions found between QTLs for cuticle phenolics. The number of QTLs with relatively small effects, together with the complexity of the interactions among them and the presence of QTLs with positive and negative effects, hampered the identification of a genetic combination that reduced the percentage of cuticle phenolics to levels similar to those of TO-937. Out of the six positive × positive QTL combinations, the only combination of TO-937 introgressions that allowed an increase in cuticle phenolics compared to the single QTL lines was *ph5.2*+*ph12.1*, while the others showed no interaction or a negative one. However, none of the double negative × negative combinations was able to reduce the percentage of cuticle phenolics beyond the single QTL lines. This could only be achieved with two triple QTL combinations *ph7.1 + 7.2*+*ph8.1* and *ph7.1 + 7.2*+*ph12.2*, none of which included *ph1.1*, the QTL with the highest additive effect on the RIL population. Indeed, a positive epistatic effect arose from the combination of *ph1.1* and *ph7.1*, since a partial cancelation of their negative effects, which deserves further study, was observed. Most of the positive × negative QTL combinations showed an intermediate behavior between the single QTL lines that, in some cases, led to a mutual cancelation of their effects; however, in five of such combinations, only the negative effect was cancelled, whereas in *ph1.1*+*ph12.1*, the opposite occurred.

### 4.2 Implications of cuticle phenolics in the improvement of desirable traits

None of the structural genes involved in phenolic acid and flavonoid biosynthesis were located within any of the phenolic QTL regions reported (Barraj Barraj et al., 2021) suggesting that the genes involved may be related to phenolic transport to the cuticle and/or vacuole, glycosylation, or regulation of the phenylpropanoid metabolic pathway, as it is the case of *MYB12*, the candidate gene for *ph1.1* (Adato et al., 2009; Barraj Barraj et al., 2021; González Moreno et al., 2023). Hence, further dissection of the phenolic fraction of the parental and single QTL lines using CRM and expression analyses of genes involved in flavonoid biosynthesis has allowed us to delve into specific characteristics of cuticle phenolic accumulation that could lead to a better approach to identify candidate genes behind these genomic regions. Hence, by targeting the inner cuticle side, the phenolic fraction accumulated during ripening could be studied. The highest reduction in *CHS* expression and chalconaringenin/phenolic acid ratio observed in *ph1.1* is in accordance with this QTL displaying the highest additive effect in the RIL population, but does not explain its moderate reduction in total cuticle phenolics (Barraj Barraj et al., 2021). Silencing *MYB12*, the candidate gene behind this QTL, altered the phenylpropanoid pathway, affecting both chalconaringenin and phenolic acid levels within the cuticle (Adato et al., 2009). This suggests that the TO-937 allele of *MYB12* has a clear effect on chalconaringenin accumulation within the cuticle, but little effect on phenolic acids, thus explaining the low reduction in total cuticle phenolics exhibited by *ph1.1*. It is possible then that the effect on phenolic acids was only measurable when *MYB12* is silenced. On the other hand, *ph7.1 + 7.2* and *ph12.2*, the QTL lines that displayed the highest negative effect on total cuticle phenolics, had a more equilibrated effect on both chalconaringenin and phenolic acids, positing that their highest reduction in total cuticle phenolics is the consequence of an effect in both phenolic fractions. A special case is *ph4.1*, a QTL with increased percentage of cuticle phenolics, a high *A*_1550_/*A*_1620_ band ratio indicative of a chalconaringenin enriched fraction, but low *CHS1* and *CHS2* expression. This seems to point to a preferential transport of chalconaringenin to the cuticle.

Identification of genomic regions that enrich the cuticle in phenolic compounds and/or increase the fraction of chalconaringenin are important for improving the thermal and mechanical properties of the cuticle. Recently, variability for the glass transition temperature (*T_g_*) in RR tomatoes has been reported, which was associated with the percentage of cuticle phenolics (Benítez et al., 2026). Thus, it was postulated that a higher percentage of cuticle phenolics increases *T_g_* due to their contribution to the hydrogen bond network within the cuticle. MM has a low *T_g_*, approximately 8°C, which implies that, above this temperature, the cuticle exhibits a mechanically weaker behavior and higher permeability (Benítez et al., 2026). On the other hand, chalconaringenin has been shown to increase cuticle’s elasticity, conferring mechanical resistance (España et al., 2014a; Benítez et al., 2026). Hence, increasing the percentage of cuticle phenolics could be a strategy to improve the mechanical resistance and water barrier properties during storage and on the vine. In this sense, molecular markers herein employed to identify QTLs could be used to introduce TO-937 genomic regions such as *ph4.1* or *ph12.1* within the domesticated tomato and generate an ideotype with a cuticle enriched in phenolic compounds for breeding purposes.

Tomato *CHS1* and *CHS2* have notable sequence similarity and comparable expression profile during ripening, which has precluded the study of their individualized effects (España et al., 2014a). The specific reduction in *CHS1* expression, without affecting *CHS2* levels, in combination with the significant effect on cuticle color observed in *ph7.1 + 7.2*, suggests that *CHS1* could be responsible for the synthesis of chalconaringenin that is transported to the cuticle. It has been reported that *CHI1* is not expressed in the peel of ripening domesticated tomatoes, leading to the accumulation of chalconaringenin in ripe fruits (Muir et al., 2001). However, *CHI1* transcript has been detected in the peel of green-fruited wild tomato species (Willits et al., 2005). Hence, *CHI1* expression during ripening in TO-937, an accession of the red-fruited species *S. pimpinellifolium*, is remarkable. This could posit that the lack of chalconaringenin in TO-937 fruit cuticle is due to the restoration of the flavonoid metabolic pathway leading to the accumulation of glycosylated flavonols in fruits, as it was reported after overexpression of a *Petunia CHI* gene in tomato (Muir et al., 2001). Thus, *CHI* expression would reduce the amount of chalconaringenin available to be transported and render the cuticle colorless. However, the high levels of *CHI1* expression during ripening in the QTL line *ph5.2*, together with the orange color of its ripe fruit cuticle, and a percentage of cuticle phenolics similar to MM, indicate otherwise. *CHI1* expression in ripening fruit peel was low compared to that of *CHS1* and *CHS2*; hence, the cuticle phenolic phenotype identified in *ph5.2* is probably due to a low rate of chalconaringenin conversion to its flavone isomer (**Figure 1C**). Nevertheless, this genomic region could be of interest to increase flavonol content in fruits without compromising the biophysical properties that chalconaringenin accumulation confers to the cuticle and could have beneficial agronomical implications. Thus, identification of candidate genes for *ph5.2* and *ph12.1* and understanding their negative interaction with *MYB12* (*ph1.1*) would not only allow us to better comprehend the regulatory pathway of phenolic accumulation but also identify strategies to improve their accumulation within the cuticle.

## Data Availability

Data generated and analyzed in this study is available in the Zenodo repository, 10.5281/zenodo.21534257.
