# Supplementary material for: QTL pyramiding uncovers epistatic interactions that modify cuticle phenolics in tomato
Source: Front Plant Sci. 2026 Aug 3;17:1863369. doi: 10.3389/fpls.2026.1863369 (PMC13478129; doi:10.3389/fpls.2026.1863369)
Supplement: Supplementary file 1 [file DataSheet1.pdf]

SUPPLEMENTARY TABLE 1 Crosses carried out between lines to accumulate QTLs for the percentage of phenolics in the cuticle. In purple/green QTLs with negative/positive effect on phenolic accumulation. (-) crosses not generated.

	ph1.1	*ph4.1*	*ph5.1*	*ph5.2*	*ph7.1+7.2*	*ph8.1*	*ph12.1*
ph1.1							
ph4.1	x						
ph5.1	x	x					
ph5.2	x	x	x				
ph7.1	x	-	-	-		-	-
ph7.2	x	-	-	-		-	-
ph7.1+7.2	x	x	x	x			
ph8.1	x	x	x	x	x		
ph12.1	x	x	x	x	x	x	
ph12.2	x	x	x	x	x	x	x

SUPPLEMENTARY TABLE 2 List of primers employed for marker assisted selection. F, forward; R, reverse; bp, base pair; T ann, temperature of annealing. In square brackets the polymorphism (snp) between 'Moneymaker' and TO-937.

Marker	QTL	Sequence	SNP	Size (bp)	T ann (ºC)
myb12_F<br>myb12_R	*ph1.1*	CCCACATTCCAAATATAA<br>TGAAGAAGCAACAACAAT	[A/C]<br>[A/C]	152	56
snp_302a_F<br>snp_302a_R	*ph4.1*	AAAGGGTTAATCGCAGTGACC<br>TAACAGGAAGAGACAAAACGGT	[T/C]	75	62
solcap_49234_F<br>solcap_49234_R	*ph5.1*	TCTTATTTGAAGGCTTGGGAAA<br>TATGCCATCCATCTGATCCA	[A/G]	75	55
solcap37577_F<br>solcap37577_R	*ph5.2*	TCACATGATCCAGCATTAGGAGA<br>GTGTTGCGTGACTCCTTTGT	[A/G]	77	58
snp_446a_F<br>snp_446a_R	*ph7.1*	AGAGGTGCCTTGAGTTGGAA<br>ACGAGATCATCACTGTGGGA	[A/G]	76	57
solcap_52557_F<br>solcap_52557_R	*ph7.2*	TCAAACACCTTTGGATCTTTCA<br>TGAGTTCACATGATTCAGTTTAC<br>G	[T/C]	91	55
solcap_48592_F<br>solcap_48592_R	*ph8.1*	CCACAGTACGTGCTTCTTGG<br>CATGCTTTTTGGAAGTTCAGAG	[A/G]	87	58
solcap_41222_F<br>solcap_41222_R	*ph12.1*	TTTGATCTGCAGCCTGAGGA<br>ACAGCAAAGTGATAGAGATTGGG	[T/C]	77	58
solcap_55706_F<br>solcap_55706_R	*ph12.2*	CGCGTATAGTGATGAAAGGAAGA<br>AACTGGTAAGGTAGGCGCAA	[A/G]	86	58

SUPPLEMENTARY TABLE 3 Primers employed for RT-qPCR. * Taken from Expósito-Rodríguez et al. (2008).

Gene symbol	Forward and Reverse Primers (5'-3')	Fragment length (bp)	Efficiency
CHS1	GGGCCTAAAGCCTGAGAAAC<br>GGCCTTTCTCATTTCATCCA	106	0.953
CHS2	ATGAGAAAGGCCTCATCCAA<br>TGGAGCACAACAGTCTCAACA	110	0.918
CHI1	AGAGAATCACGTGTTCCCAC<br>TTCCAAGTACACACCGATCG	139	0.978
*CAC**	CCTCCGTTGTGATGTAACTGG<br>ATTGGTGGAAAGTAACATCATCG	173	0.953
*SAND**	TTGCTTGGAGGAACAGACG<br>GCAAACAGAACCCCTGAATC	164	0.944
*EXP**	GCTAAGAACGCTGGACCTAATG<br>TGGGTGTGCCTTTCTGAATG	183	0.959

SUPPLEMENTARY TABLE 4 Band assignation within the 1680-1480 cm^{-1} spectral region according to the literature.

Wavenumber (cm^{-1})	Assignment	Cuticle component	References
1550	C-O stretching<br>O-H bending	Flavonoids	González Moreno et al. (2021)
1585	*v*(C-C) aromatic	Phenolic acids<br>Flavonoids	Ramírez et al. (1992)<br>Brandán et al. (2010)<br>Swisłocka et al. (2012)<br>Prats Mateu et al. (2016)
1605	Aromatic C = C stretching<br>Vinyl C-C stretching<br>C-H bending	Phenolic acids<br>Flavonoids	González Moreno et al. (2021)<br>Prats Mateu et al. (2016)<br>Heredia-Guerrero et al. (2014)
1620	Aromatic C = C stretching	Mainly phenolic acids	González Moreno et al. (2023)

References

Brandán, S.A., Márquez López, F., Montejo, M., López González, J.J., Ben Altabef, A. (2010). Theoretical and experimental vibrational spectrum study of 4-hydroxybenzoic acid as monomer and dimer. *Spectrochim. Acta A Mol. Biomol. Spectrosc.* 75, 1422-1434. doi: 10.1016/j.saa.2010.01.012.

González Moreno, A., Prieto, P., Ruiz Delgado, M.C., Domínguez, E., Heredia, A., De Cózar, A. (2021). Structure, isomerization and dimerization processes of naringenin flavonoids. *Phys. Chem. Chem. Phys.* 23, 18068-18077. doi: 10.1039/D1CP01161H.

González Moreno, A., Domínguez, E., Mayer, K., Xiao, N., Bock, P., Heredia, A., Gierlinger, N. (2023). 3D (x-y-t) Raman imaging of tomato fruit cuticle: microchemistry during development. *Plant Physiol.* 191, 219-232. doi: 10.1093/plphys/kiac369.

Heredia-Guerrero, J.A., Benítez, J.J., Domínguez, E., Bayer, I., Cingolani, R., Athanassiou, A., Heredia, A. (2014). Infrared and Raman spectroscopic features of plant cuticles: a review. *Front. Plant Sci.* 5, 305. doi: 10.3389/fpls.2014.00305.

Prats Mateu, B.P., Hauser, M.T., Heredia, A., Gierlinger, N. (2016). Waterproofing in Arabidopsis: following phenolics and lipids in situ by Confocal Raman microscopy. *Front. Chem.* 4, 10. doi: 10.3389/fchem.2016.00010.

Ramírez, F.J., Luque, P., Heredia, A., Bukovac, M.J. (1992). Fourier transform IR study of enzymatically isolated tomato fruit cuticular membrane. *Biopolymers* 32, 1425-1429. doi: 10.1002/bip.360321102.

Swisłocka, R., Kowczyk-Sadowy, M., Kalinowska, M., Lewandowski, W. (2012). Spectroscopic (FT-IR, FT-Raman, ^{1}H and ^{13}C-NMR) and theoretical studies of p-coumaric acid and alkali metal *p*-coumarates. *Spectroscopy* 27, 35-48. doi: 10.3233/SPE-2012-0568.

SUPPLEMENTARY TABLE 5 Isolated fruit cuticle color at red ripe of the parental lines and single QTL-lines. CIE L*C*h° space was employed. In purple/green QTLs with negative/positive effect on phenolic accumulation. Data presented as means ± SE. Asterisks indicate significant differences with MM control at *P* < 0.05 *, *P* < 0.01 ** and *P* < 0.001 ***.

Lines	L*	C*	h°
MM	74.73 ± 0.24	79.84 ± 0.21	83.45 ± 0.28
TO-937	84.87 ± 0.21***	46.17 ± 1.03***	100.68 ± 0.45***
ph1.1	80.81 ± 0.26***	74.23 ± 0.73***	91.88 ± 0.37***
ph4.1	74.49 ± 0.23	82.35 ± 0.35***	81.51 ± 0.33***
ph5.1	75.62 ± 0.35	81.13 ± 0.33	84.52 ± 0.46
ph5.2	75.80 ± 0.38*	81.03 ± 0.28	84.05 ± 0.55
ph7.1+7.2	82.23 ± 0.20***	72.60 ± 0.65***	92.80 ± 0.32***
ph8.1	80.27 ± 0.30***	76.20 ± 0.50***	91.20 ± 0.35***
ph12.1	73.92 ± 0.19	81.26 ± 0.26	82.36 ± 0.23
ph12.2	79.68 ± 0.25***	75.27 ± 0.43***	90.78 ± 0.31***

SUPPLEMENTARY TABLE 6 Percentage of cuticle phenolics for the parental and single QTL-lines across different seasons. In purple/green QTLs with negative/positive effect on phenolic accumulation. Data presented as means ± SE. Asterisks indicate significant differences with MM control at $P < 0.05$ *, $P < 0.01$ ** and $P < 0.001$ ***.

Lines	Summer 1	Summer 2	Winter
MM	6.0 ± 0.1	6.0 ± 0.2	4.4 ± 0.1
TO-937	1.2 ± 0.1***	1.2 ± 0.1***	1.3 ± 0.1***
ph1.1	4.2 ± 0.1***	4.6 ± 0.1***	3.5 ± 0.3**
ph4.1	8.0 ± 0.2***	8.0 ± 0.3***	7.6 ± 0.1***
ph5.1	6.2 ± 0.2	6.3 ± 0.1	4.9 ± 0.1
ph5.2	6.4 ± 0.1	6.2 ± 0.1	4.5 ± 0.2
ph7.1+7.2	3.5 ± 0.2***	3.4 ± 0.1***	2.5 ± 0.1***
ph8.1	4.8 ± 0.1***	5.0 ± 0.1**	3.5 ± 0.1**
ph12.1	7.5 ± 0.2***	7.4 ± 0.2***	6.0 ± 0.3***
ph12.2	3.6 ± 0.1***	3.2 ± 0.1***	2.8 ± 0.1***

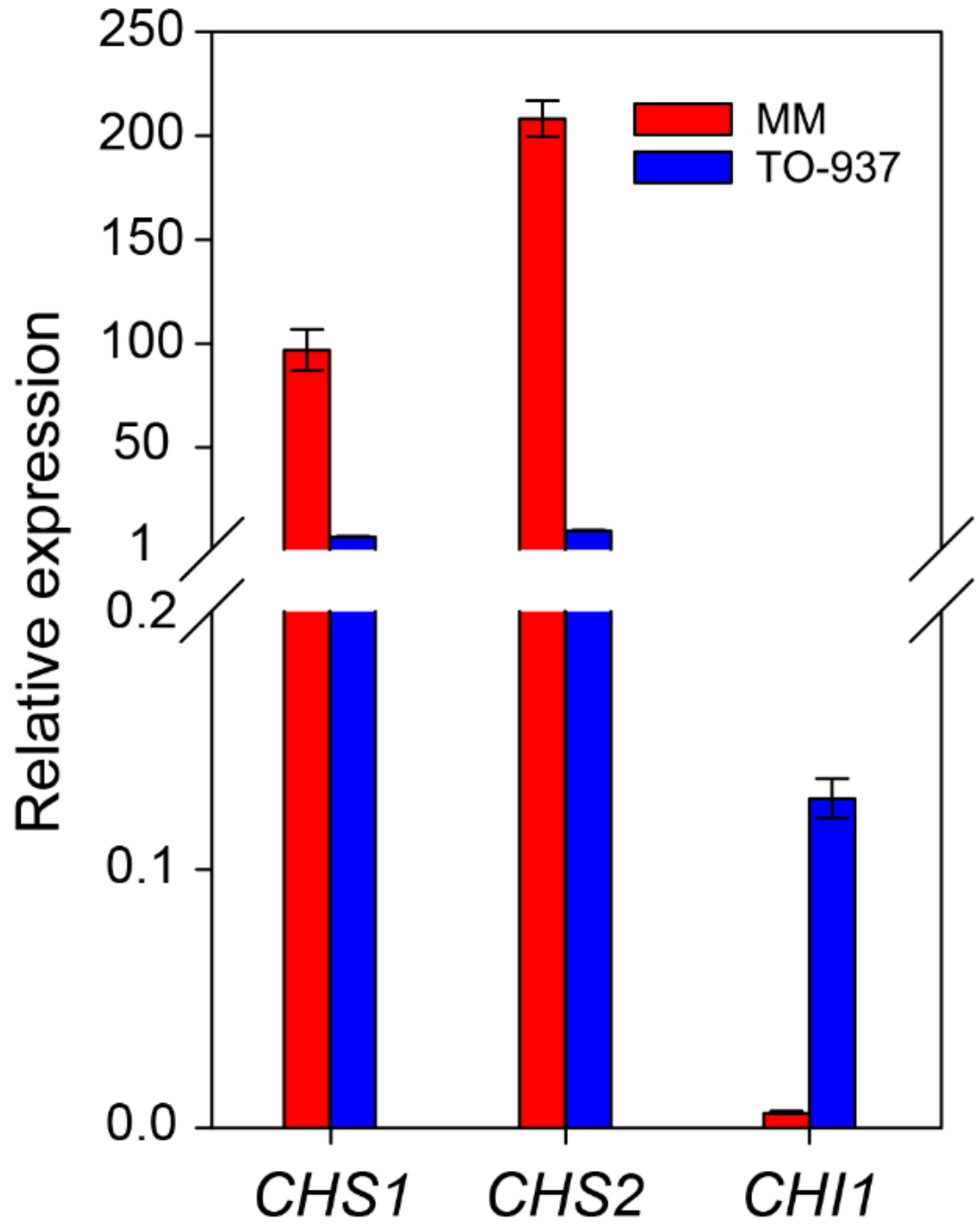

SUPPLEMENTARY FIGURE 1

Relative expression levels of genes involved in the biosynthesis of flavonoids. Gene expression was carried out in the epicarp at breaker of parental lines MM and TO-937. Data are expressed as means ± SE. *CHS*, *CHALCONE SYNTHASE*; *CHI, CHALCONE ISOMERASE*.

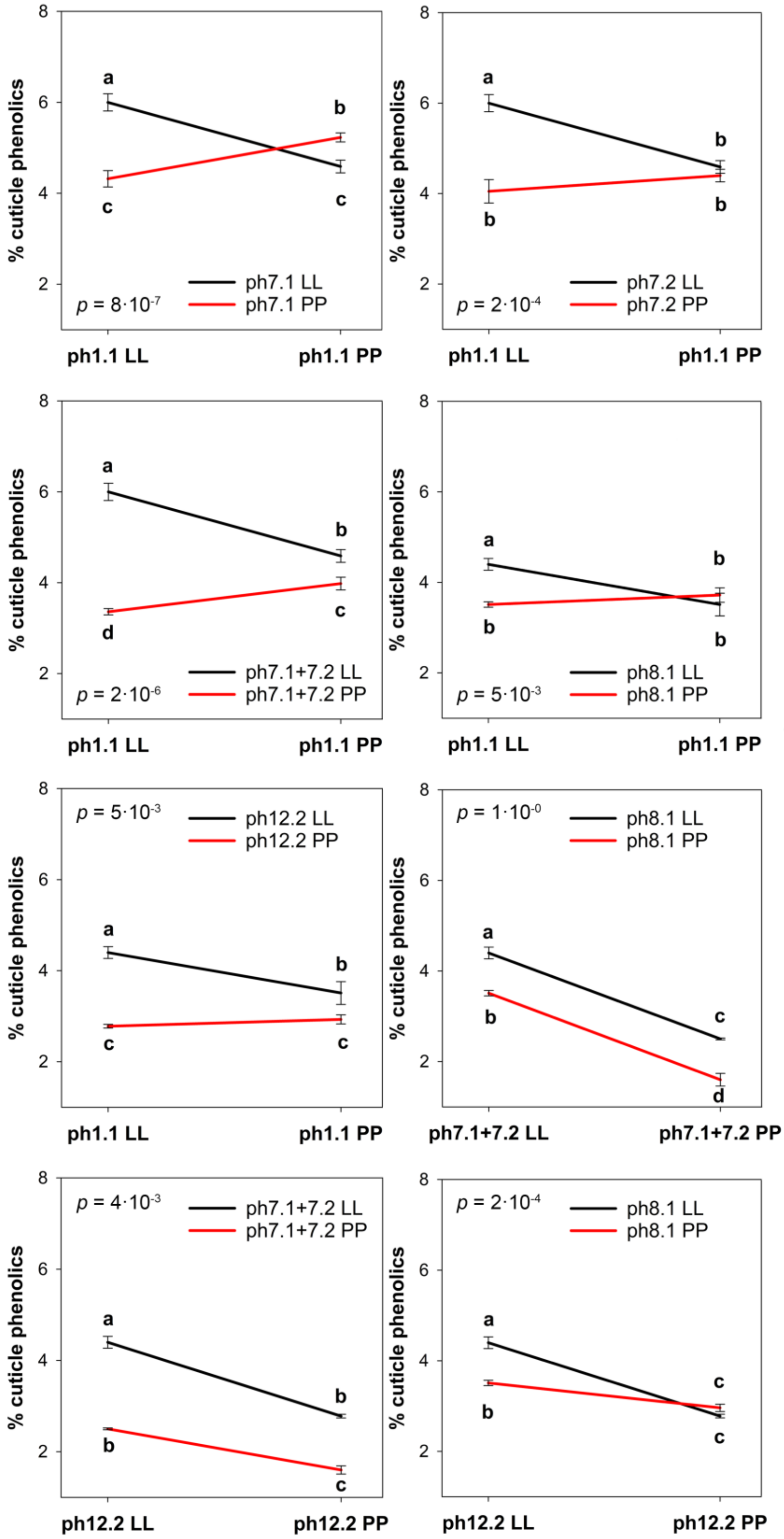

SUPPLEMENTARY FIGURE 2

Epistatic interactions between QTLs with negative effect on cuticle phenolics at red ripe. Data presented as means ± SE. Letters indicate significant differences among lines harboring different QTLs (P < 0.05). p-values associated with epistatic interaction are also given.

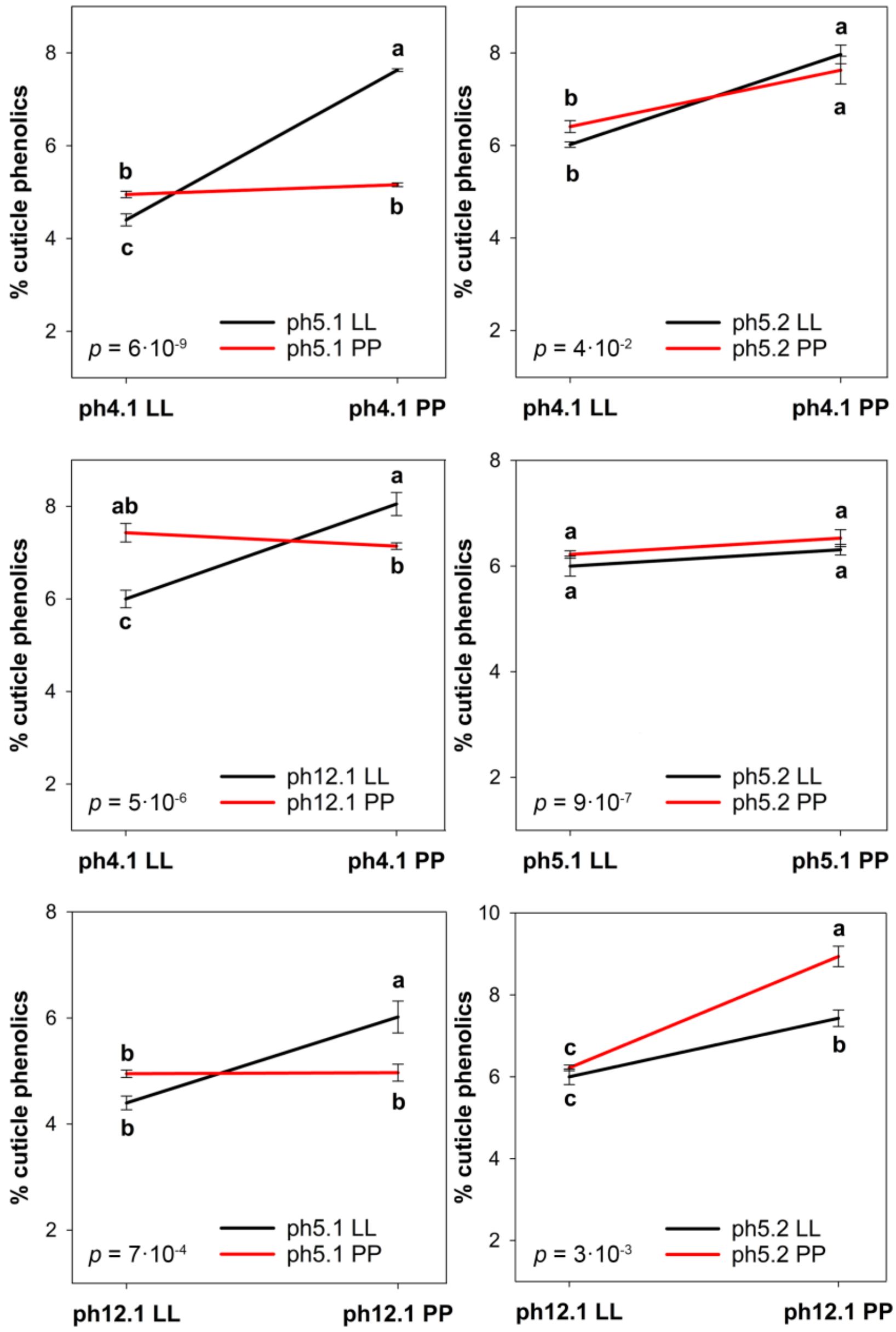

SUPPLEMENTARY FIGURE 3

Epistatic interactions between QTLs with positive effect on cuticle phenolics at red ripe. Data presented as means ± SE. Letters indicate significant differences among lines harboring different QTLs (*P* < 0.05). p-values associated with epistatic interaction are also given.

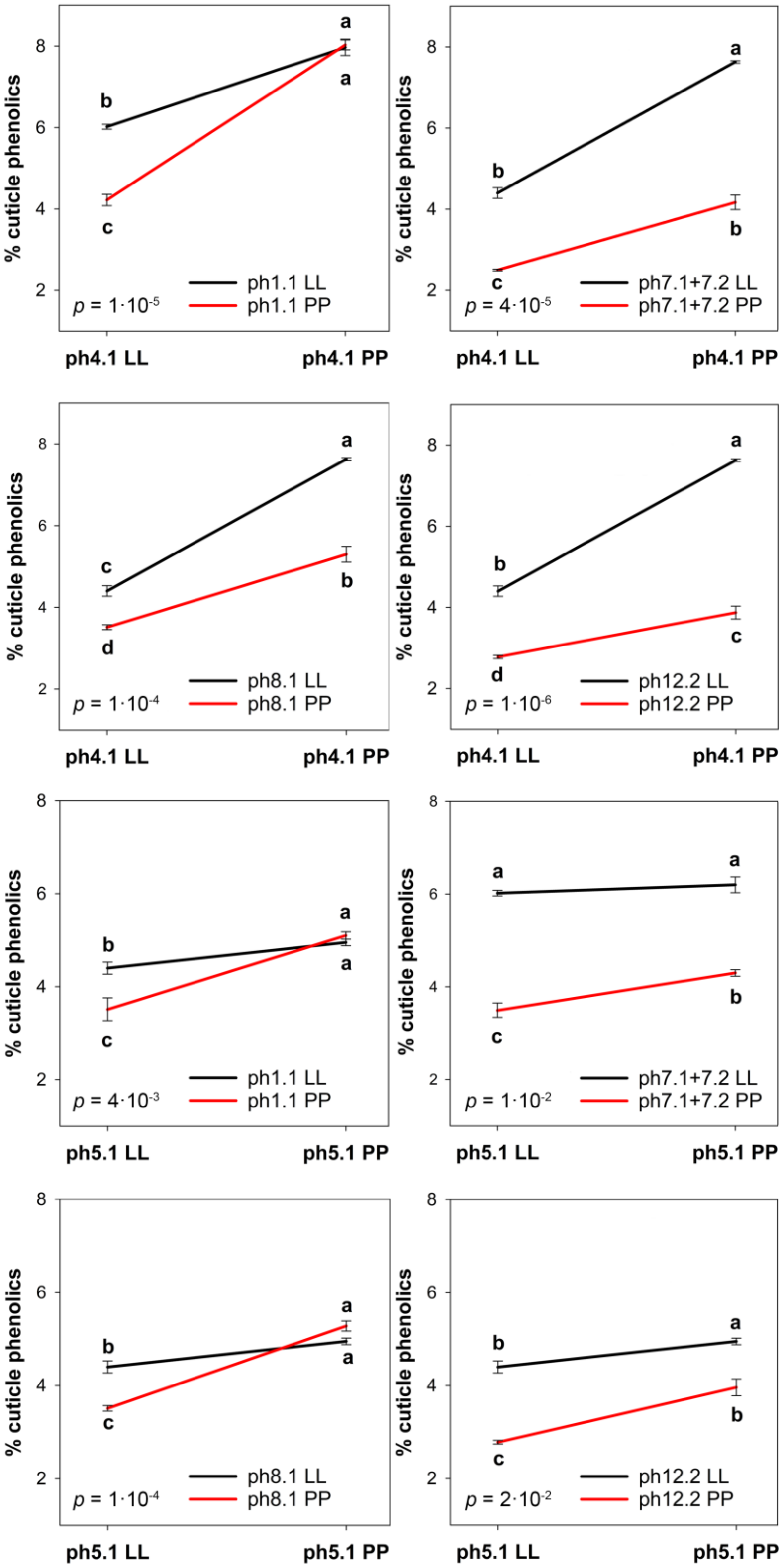

SUPPLEMENTARY FIGURE 4

Epistatic interactions between QTLs with positive and negative effect on cuticle phenolics at red ripe. Data presented as means ± SE. Letters indicate significant differences among lines harboring different QTLs ($P < 0.05$). p-values associated with epistatic interaction are also given.

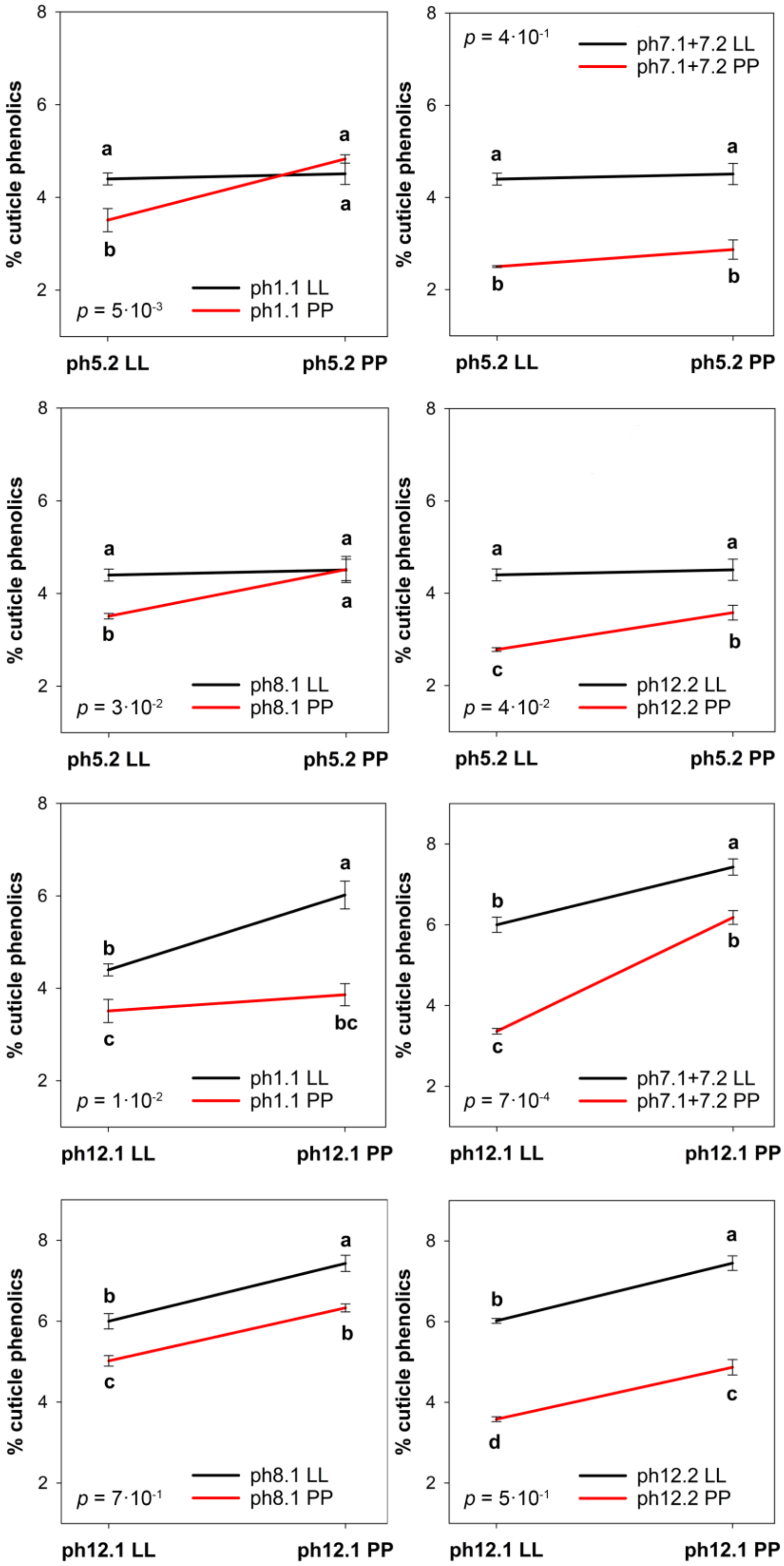

SUPPLEMENTARY FIGURE 5

Epistatic interactions between QTLs with positive and negative effect on cuticle phenolics at red ripe. Data presented as means ± SE. Letters indicate significant differences among lines harboring different QTLs ($P < 0.05$). p-values associated with epistatic interaction are also given.

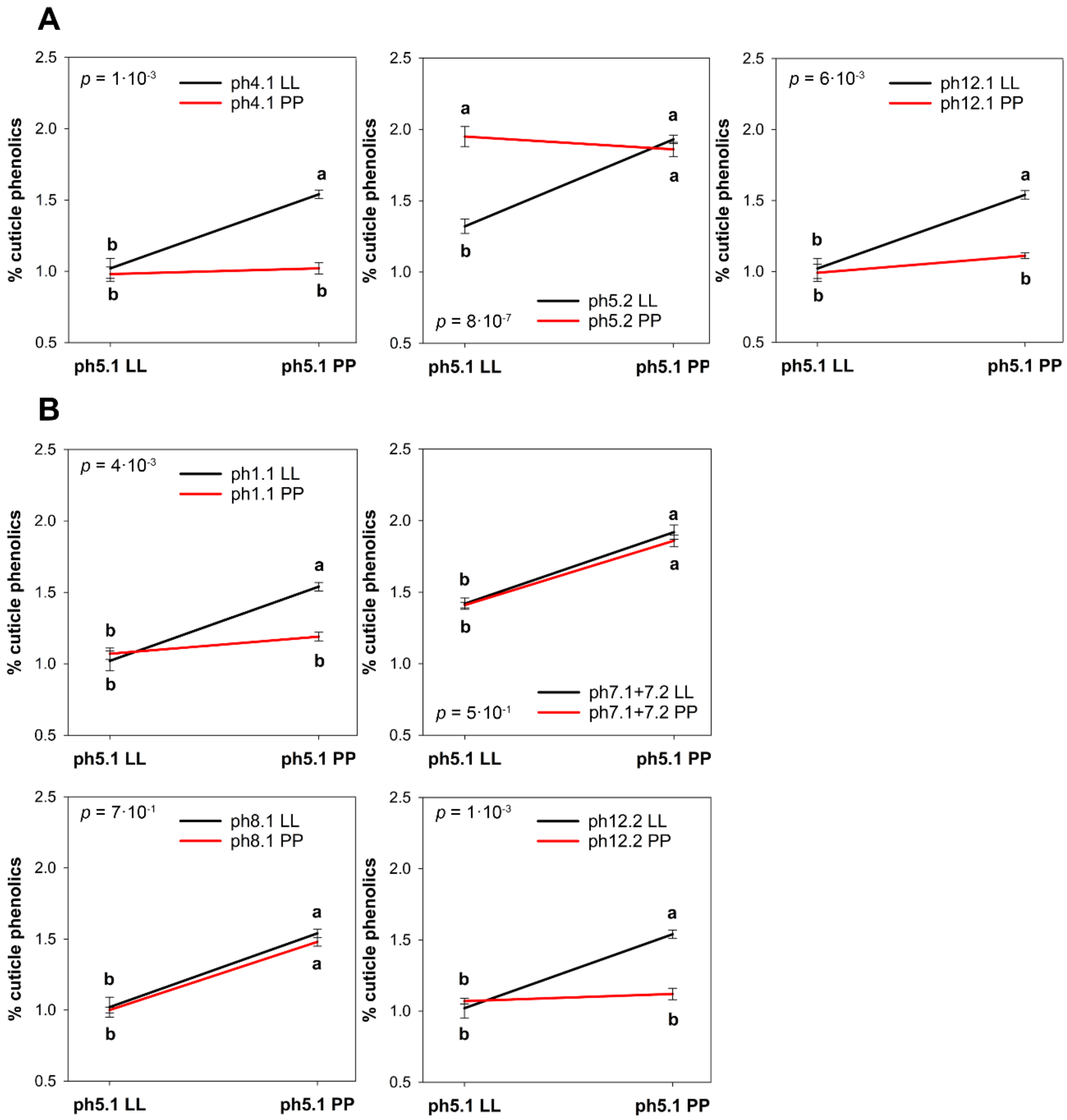

SUPPLEMENTARY FIGURE 6

Epistatic interactions between *ph5.1* and the rest of QTLs for cuticle phenolics at immature green. (A) Interaction with QTLs of positive effect. (B) Interactions with QTLs displaying a negative effect. Data presented as means ± SE. Letters indicate significant differences among lines harboring different QTLs ($P < 0.05$). p-values associated with epistatic interaction are also given.

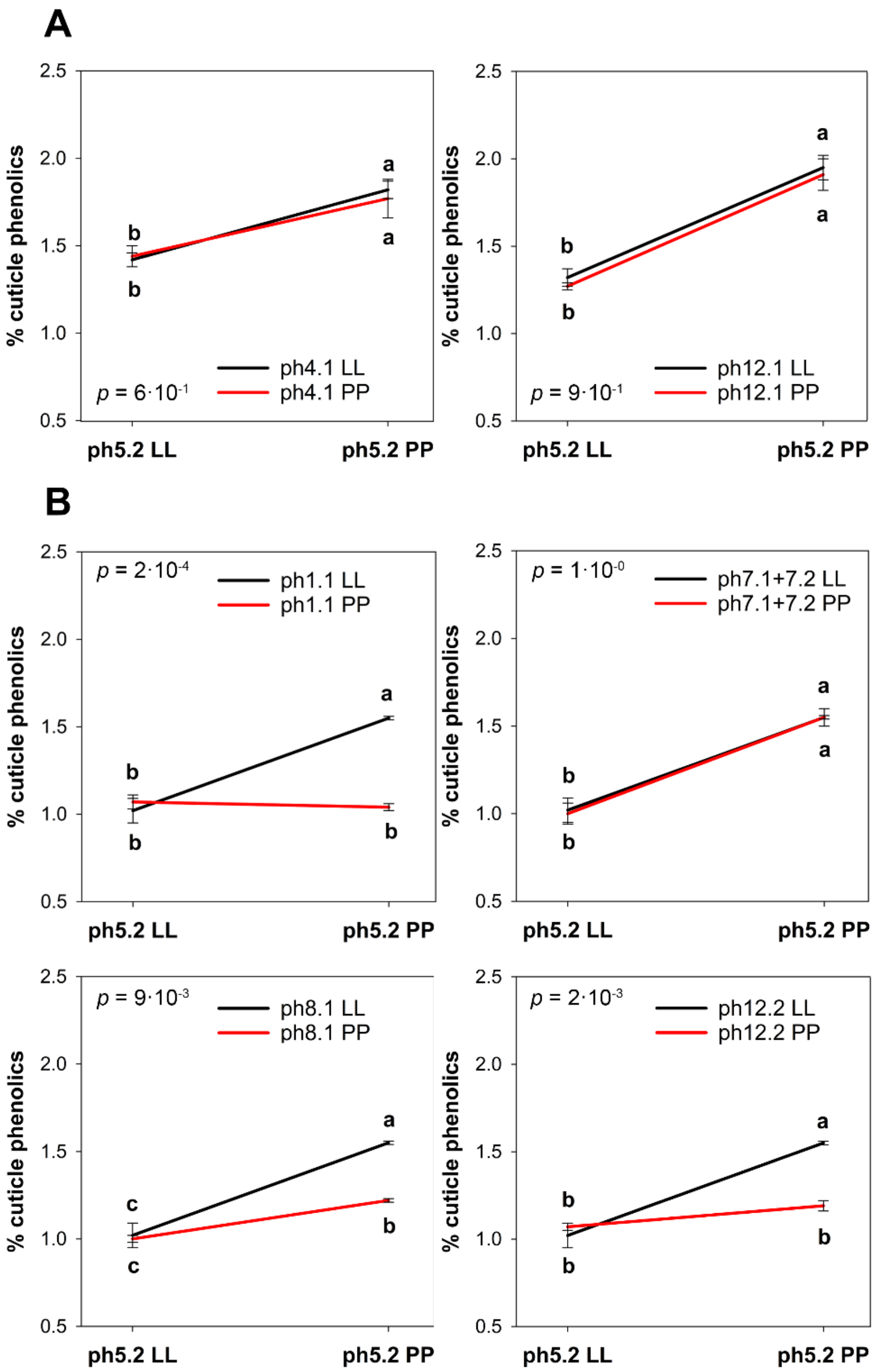

SUPPLEMENTARY FIGURE 7

Epistatic interactions between *ph5.2* and the rest of QTLs for cuticle phenolics at immature green. (A) Interaction with QTLs of positive effect. (B) Interactions with QTLs displaying a negative effect. Data presented as means ± SE. Letters indicate significant differences among lines harboring different QTLs ($P < 0.05$). p-values associated with epistatic interaction are also given.

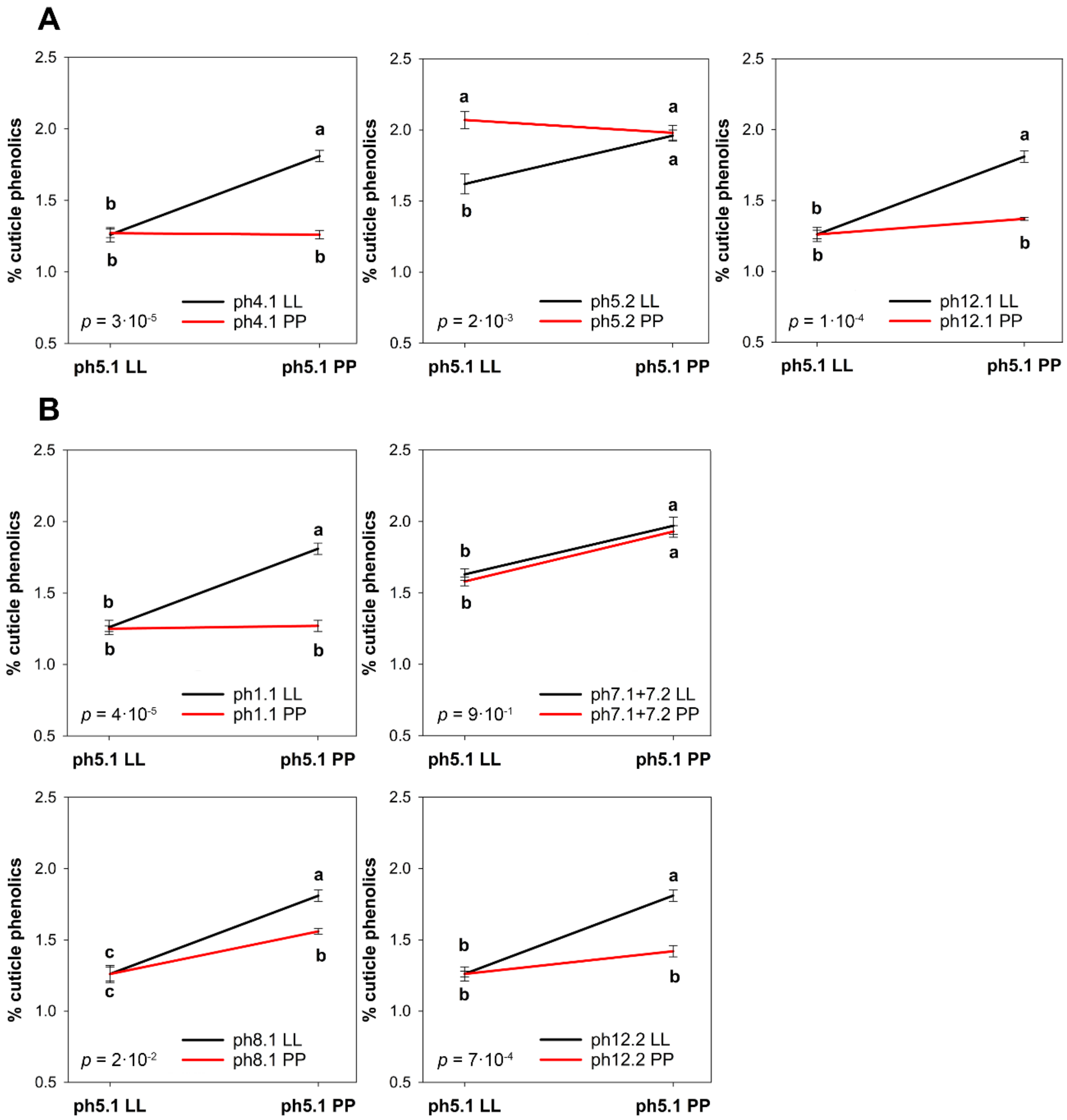

SUPPLEMENTARY FIGURE 8

Epistatic interactions between *ph5.1* and the rest of QTLs for cuticle phenolics at mature green. (A) Interaction with QTLs of positive effect. (B) Interactions with QTLs displaying a negative effect. Data presented as means ± SE. Letters indicate significant differences among lines harboring different QTLs ($P < 0.05$). p-values associated with epistatic interaction are also given.

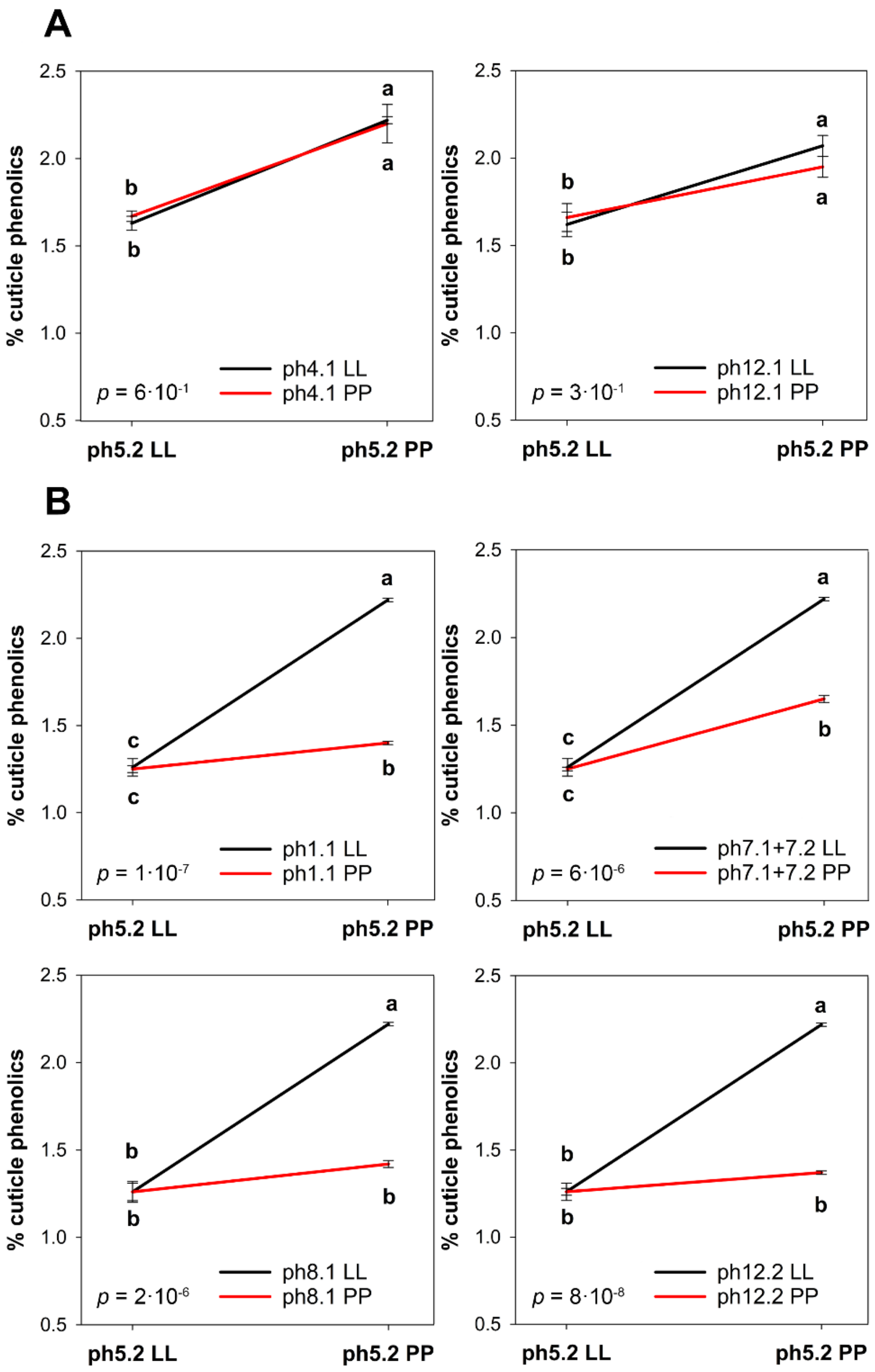

SUPPLEMENTARY FIGURE 9

Epistatic interactions between *ph5.2* and the rest of QTLs for cuticle phenolics at mature green. (A) Interaction with QTLs of positive effect. (B) Interactions with QTLs displaying a negative effect. Data presented as means ± SE. Letters indicate significant differences among lines harboring different QTLs ($P < 0.05$). p-values associated with epistatic interaction are also given.

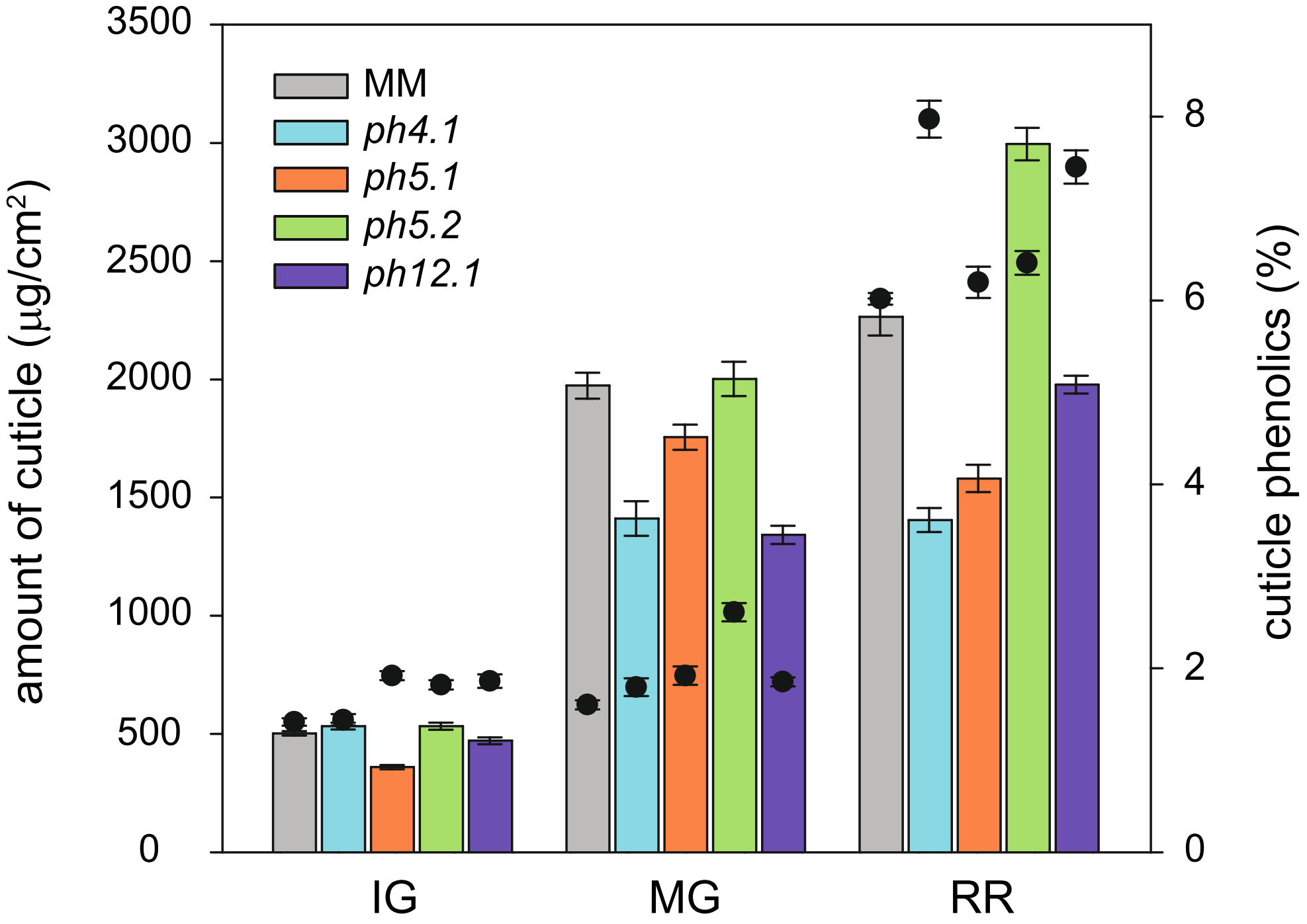

SUPPLEMENTARY FIGURE 10

Amount of cuticle and percentage of cuticle phenolics in MM and the ILs harboring QTLs for positive effect on phenolics. Bars represent the amount of cuticle and scattered dots the percentage of phenolics. Data are presented as means ± SE. IG, immature green; MG, mature green; RR, red ripe.

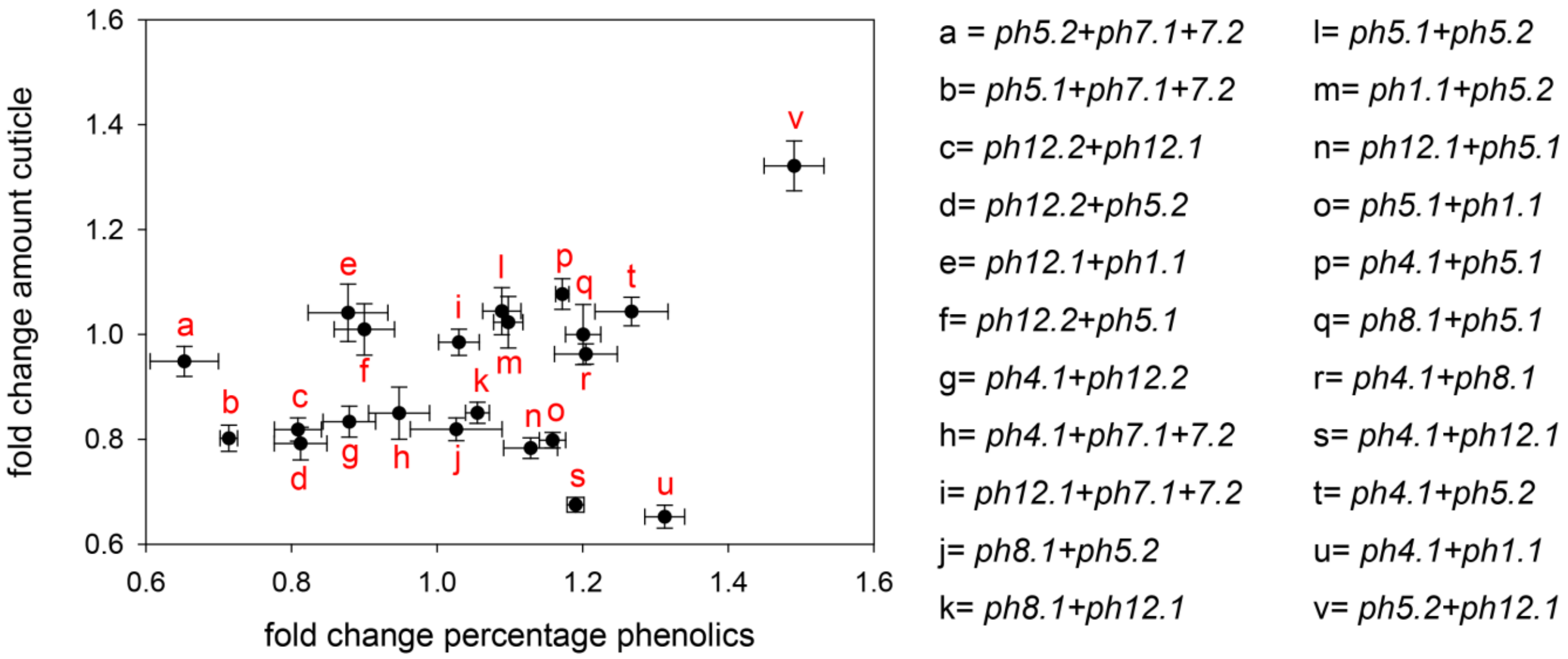

SUPPLEMENTARY FIGURE 11

Fold change relative to MM control for the amount of cuticle and percentage of phenolics. Data shown corresponds to QTL combinations containing *ph4.1*, *ph5.1*, *ph5.2* or *ph12.1*, which coincided with QTLs for the amount of cuticle. Data presented as mean ± SE.

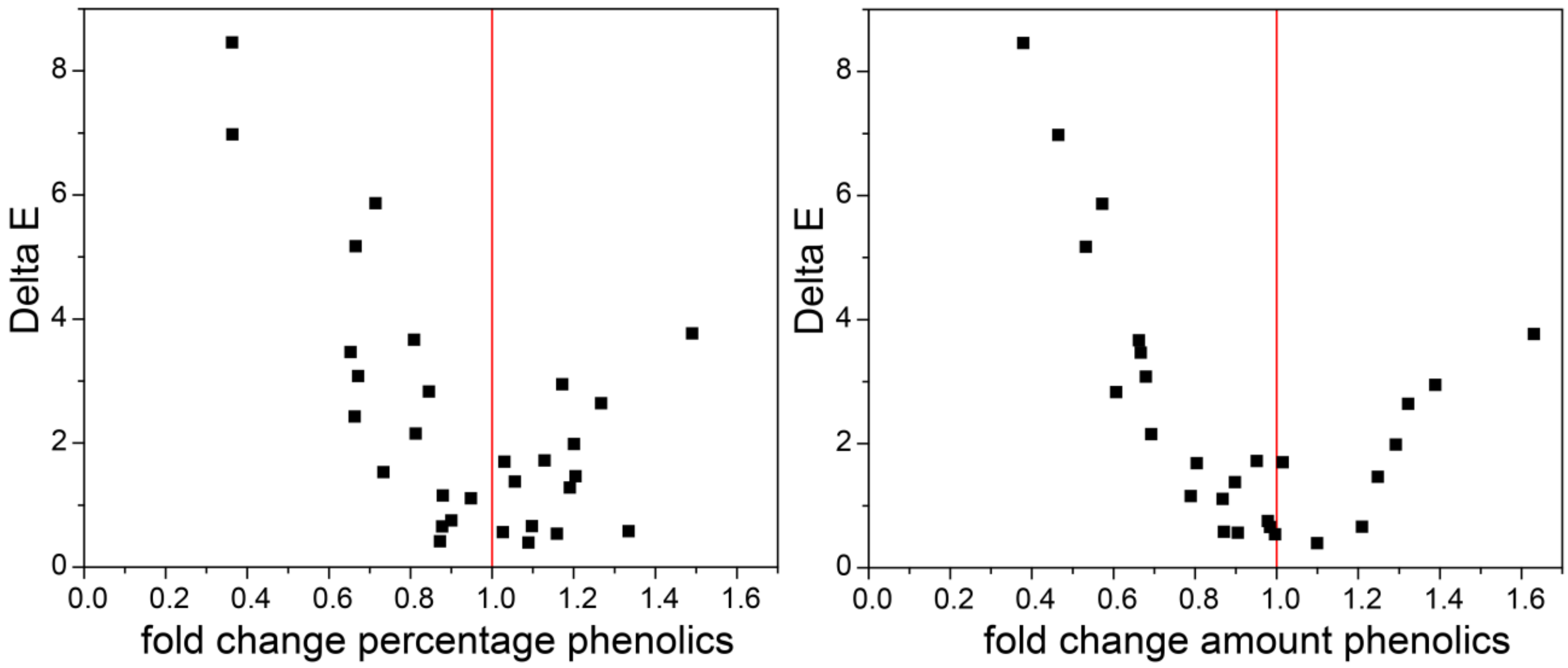

SUPPLEMENTARY FIGURE 12

Relationship between color difference and fold change in cuticle phenolics. Data are shown relative to MM control for percentage and amount of cuticle phenolics in the different lines accumulating QTLs. Delta E, color difference relative to MM control using CIE DE 2000 equation. Red line separates values in percentage or amount of cuticle phenolics below and above MM control.
